# Wavelength-Dependent Solar N_2_ Fixation into Ammonia and Nitrate in Pure Water

**DOI:** 10.34133/2020/3750314

**Published:** 2020-05-29

**Authors:** Wenju Ren, Zongwei Mei, Shisheng Zheng, Shunning Li, Yuanmin Zhu, Jiaxin Zheng, Yuan Lin, Haibiao Chen, Meng Gu, Feng Pan

**Affiliations:** ^1^School of Advanced Materials, Peking University, Shenzhen Graduate School, China; ^2^School of Advance Manufacturing Engineering, Chongqing University of Posts and Telecommunications, Chongqing, China; ^3^Department of Materials Science and Engineering, Southern University of Science and Technology, China; ^4^SUSTech Academy for Advanced Interdisciplinary Studies, Southern University of Science and Technology, China; ^5^Institute of Chemistry, Chinese Academy of Sciences, Beijing, China

## Abstract

Solar-driven N_2_ fixation using a photocatalyst in water presents a promising alternative to the traditional Haber-Bosch process in terms of both energy efficiency and environmental concern. At present, the product of solar N_2_ fixation is either NH_4_^+^ or NO_3_^−^. Few reports described the simultaneous formation of ammonia (NH_4_^+^) and nitrate (NO_3_^−^) by a photocatalytic reaction and the related mechanism. In this work, we report a strategy to photocatalytically fix nitrogen through simultaneous reduction and oxidation to produce NH_4_^+^ and NO_3_^−^ by W_18_O_49_ nanowires in pure water. The underlying mechanism of wavelength-dependent N_2_ fixation in the presence of surface defects is proposed, with an emphasis on oxygen vacancies that not only facilitate the activation and dissociation of N_2_ but also improve light absorption and the separation of the photoexcited carriers. Both NH_4_^+^ and NO_3_^−^ can be produced in pure water under a simulated solar light and even till the wavelength reaching 730 nm. The maximum quantum efficiency reaches 9% at 365 nm. Theoretical calculation reveals that disproportionation reaction of the N_2_ molecule is more energetically favorable than either reduction or oxidation alone. It is worth noting that the molar fraction of NH_4_^+^ in the total product (NH_4_^+^ plus NO_3_^−^) shows an inverted volcano shape from 365 nm to 730 nm. The increased fraction of NO_3_^−^ from 365 nm to around 427 nm results from the competition between the oxygen evolution reaction (OER) at W sites without oxygen vacancies and the N_2_ oxidation reaction (NOR) at oxygen vacancy sites, which is driven by the intrinsically delocalized photoexcited holes. From 427 nm to 730 nm, NOR is energetically restricted due to its higher equilibrium potential than that of OER, accompanied by the localized photoexcited holes on oxygen vacancies. Full disproportionation of N_2_ is achieved within a range of wavelength from ~427 nm to ~515 nm. This work presents a rational strategy to efficiently utilize the photoexcited carriers and optimize the photocatalyst for practical nitrogen fixation.

## 1. Introduction

Ammonia (NH_3_) and nitrate are widely used for agricultural and chemical synthesis purposes [1–4]. Due to the environmental issues and energy crisis in recent years, NH_3_ has also gained growing interest as a liquid fuel for fuel cells due to its high energy density and easy storage [5]. However, the industrial production of NH_3_ is mainly based on the traditional Haber-Bosch process, which consumes nearly 2% of global energy and emits about 1% of greenhouse gases [6, 7]. Extra energy is needed for the production of nitrate from NH_3_ [1, 8]. As a green and environmentally friendly alternative for ammonia and nitrate synthesis, solar-driven nitrogen fixation in aqueous media using a photocatalyst at room temperature and atmospheric pressure presents a tantalizing approach [9–13]. However, the current efficiency of synthesizing NH_3_ and nitrate (NO_3_^−^) by a photocatalytic approach is still far from practical purpose [2].

Either NH_4_^+^ or NO_3_^−^ as a solar N_2_ fixation product has been reported based on a tungsten oxide photocatalyst [8, 14], in which only the photogenerated electrons or holes are utilized. Few studies have exhibited the simultaneous coproduction of NH_4_^+^ and NO_3_^−^ and a mechanism of wavelength-dependent solar N_2_ fixation. This process utilizes both photogenerated electrons and holes more efficiently. During the N_2_ reduction reaction (NRR) process, oxygen evolution reaction (OER) will participate in pure water without sacrificial reagents, which occurs throughout the photocatalytic N_2_ fixation process and consumes the photoexcited holes. Therefore, the overall reaction is as follows:

(1) $$\begin{array}{c} 2{\text{N}}_{2}+6{\text{H}}_{2}\text{O}\to 4\text{N}{\text{H}}_{3}+3{\text{O}}_{2} \end{array}$$

The voltage per electron is 1.13 V for the above reaction. Previous work has indicated that N_2_ can also be oxidized to NO_3_^−^ over pothole-rich ultrathin WO_3_ nanosheets [8]. It is reasonable that NO_3_^−^ and NH_3_ could be produced simultaneously, with N_2_ fixation proceeding through the following reactions:

(2) $$\begin{array}{c} 5{\text{N}}_{2}+6{\text{H}}_{2}\text{O}\to 4\text{N}{\text{H}}_{3}+6\text{NO} \end{array}$$

(3) $$\begin{array}{c} 4\text{NO}+3{\text{O}}_{2}+2{\text{H}}_{2}\text{O}\to 4\text{HN}{\text{O}}_{3} \end{array}$$

The voltage per electron is 1.57 V for Reaction (2), meaning that this reaction route is thermodynamically unfavorable as compared to Reaction (1). However, nitrogen fixation on defected surfaces can be controlled by the reaction kinetics on the catalytic sites like oxygen vacancies on the surfaces of transition metal oxides. It should be noted that Reaction (3), i.e., the oxidation of NO, can occur spontaneously in aqueous solution [15]. Since the oxygen in Reaction (3) can only come from Reaction (1) when the external O_2_ is removed from the reaction system, the maximization of NO_3_^−^ will correspond to the consumption of all produced oxygen, which gives the overall reaction as follows:

(4) $$\begin{array}{c} 4{\text{N}}_{2}+9{\text{H}}_{2}\text{O}\to 5\text{N}{\text{H}}_{3}+3\text{HN}{\text{O}}_{3} \end{array}$$

It is an established understanding that the rate-determining step for N_2_ fixation is the activation and dissociation of the extremely stable N≡N triple bond (bond strength of ~941 kJ mol^−1^) [14, 16, 17]. A key step for effective photocatalytic N_2_ fixation is to efficiently transfer the energetic photoexcited electrons to the rather inert N_2_ molecule [8]. The N≡N triple bond can be weakened and activated when electrons are injected from the solid-state catalysts into the empty antibonding *π*^∗^-orbitals of the nitrogen molecule [8, 18]. For this purpose, abundant active sites with localized electrons should be created so that the N_2_ molecule can be chemisorbed for facile electron access. Such sites serve as the effective bridge between the energetic photoelectrons and the nitrogen molecule [14]. Surface vacancies with rich localized electrons due to the charge-transfer phenomenon [19] can effectively activate and weaken the N≡N triple bond by inducing chemisorption and electron injection [2, 20, 21]. Diversified surface vacancies, including oxygen [8, 9, 12, 14, 18], sulfur [22, 23], and nitrogen [24–26], have been proven to promote the photocatalytic N_2_ fixation efficiency. On the other hand, the appropriate anion vacancies in the bulk and on the surfaces of semiconductor photocatalysts can facilitate the separation and migration of the photogenerated electrons and holes [27], which is also crucial for photocatalytic reactions [9, 12, 18]. The defects and disordered surfaces of a semiconductor alter the electronic structures by forming midgap states or band tail states [28–30], thus extending the light absorption spectrum and resulting in enhanced light absorption capability.

In this work, ultrathin W_18_O_49_ nanowires with distorted surface structures containing abundant surface oxygen vacancies were synthesized using a simple solvothermal method and are intended as a prototype for studying the wavelength-controlled N_2_ fixation in the presence of surface defects. The as-synthesized sample showed photocatalytic activity for N_2_ fixation to NH_4_^+^ and NO_3_^−^ in pure water from ultraviolet up to near the end of visible light (730 nm) and exhibited high performance under simulated solar light (AM 1.5G) irradiation. The quantum efficiency (QE) reached about 9% at 365 nm through the simultaneous generation of both NH_4_^+^ and NO_3_^−^. Both experimental and theoretical results suggested that surface oxygen vacancies serve as catalytic sites and are essential for the high efficiency of N_2_ fixation. The oxidation of N_2_ was found to be retarded at either sufficiently short or sufficiently long wavelengths, and full disproportionation of N_2_ through Reaction (4) is achieved during wavelength from ~427 nm to ~515 nm. A mechanism for wavelength-controlled N_2_ fixation via its simultaneous reduction and oxidation on defected surfaces was proposed, which sheds new light on the understanding of photocatalytic nitrogen fixation with different product selectivity and could provide guidelines for the design of future photocatalysts with higher utilization efficiency of photoexcited carriers.

## 2. Results and Discussion

**Figure 1:**
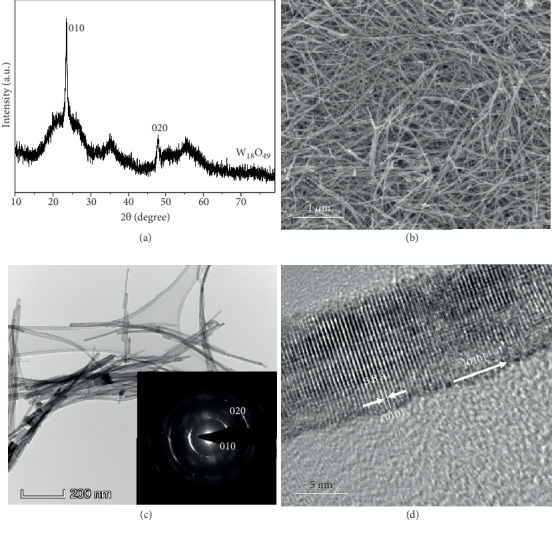
Structural characterizations of the as-synthesized W_18_O_49_ nanowires. (a) The XRD pattern. (b) The SEM image. (c) The TEM image with the SAED pattern (inset). (d) The high-resolution TEM (HRTEM) image demonstrating the (010) lattice.

W_18_O_49_ nanowires were prepared using a solvothermal method, and a reference sample was prepared by subsequently annealing the as-synthesized W_18_O_49_ nanowires at 300°C in air for 30 min to eliminate oxygen vacancies from the surface. The crystal structure and phase purity of the as-synthesized blue velvet-like product (Figure 1(a)) were revealed by X-ray diffraction (XRD) to be consistent with the standard monoclinic W_18_O_49_ (P2/m) (PDF 05-0392) as previously reported [31–33]. No visible changes in XRD patterns can be observed after annealing (Figure S1). The scanning electron microscopy (SEM) images indicate that the as-synthesized W_18_O_49_ consists of ultrathin nanowires (Figure 1(b)), while the transmission electron microscopy (TEM) image (Figure 1(c)) further confirms that the diameters and the lengths of the as-synthesized nanowires are <10 nm and >2 *μ*m, respectively. The selected area electron diffraction (SAED) pattern demonstrates that the diffraction rings belong to the (010, 020) planes of the W_18_O_49_ structure (inset in Figure 1(c)), consistent with XRD. The interplanar spacing of the (010) planes is ~3.8 Å, and the nanowire grows along the [010] direction (Figure 1(d)). After annealing, the nanowire becomes shorter and thicker (Figures S2a and S2b); however, the interplanar spacing remains 3.8 Å (Figure S2c), which further confirms that the crystal structure remains unchanged after annealing.

**Figure 2:**
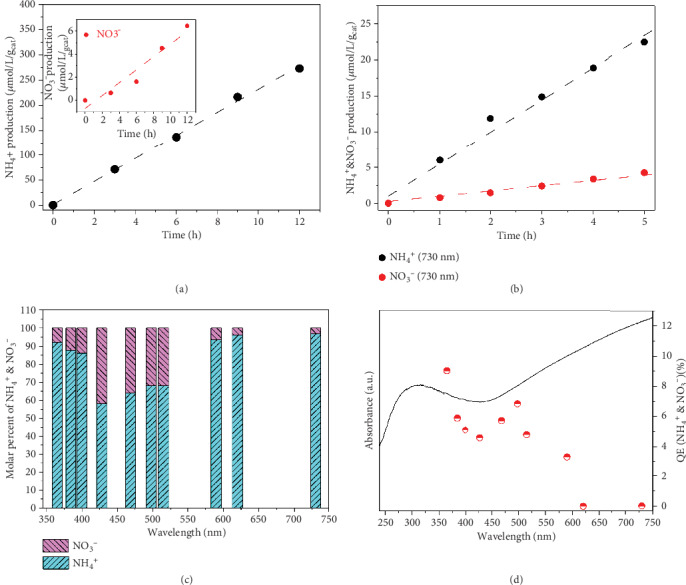
Photocatalytic performance for N_2_ fixation of the as-synthesized W_18_O_49_ nanowires under different light irradiation. (a) Solar simulator (AM 1.5G, 100 mW/cm^2^). (b) 730 nm LED (2.95 mW/cm^2^). (c) Molar percentage of NH_4_^+^ and NO_3_^−^ under the irradiation of different wavelength light. (d) The CQE of the as-synthesized W_18_O_49_ nanowires under monochromatic light irradiation along with the light absorption spectra.

The photocatalytic nitrogen fixation performance of the W_18_O_49_ nanowires under simulated solar irradiation (AM 1.5G, 400 nm-1100 nm) is presented in Figure 2(a). The yield rates of NH_4_^+^ and NO_3_^−^ within 12 h were about 22.8 *μ*mol L^−1^ g_cat_^−1^ h^−1^ and 0.54 *μ*mol L^−1^ g_cat_^−1^ h^−1^, respectively. W_18_O_49_ nanowires were also tested under irradiation of a 300 W xenon light (872 mW/cm^2^). The average NH_3_ production rate was about 65.2 *μ*mol L^−1^ g_cat_^−1^ h^−1^, and the yield rate of NO_3_^−^ was nearly 0.57 *μ*mol L^−1^ g_cat_^−1^ h^−1^ for the as-synthesized W_18_O_49_ nanowires (Figure S3a). In comparison, the average NH_3_ production rate decreased to ~1.6 *μ*mol L^−1^ g_cat_^−1^ h^−1^, and no NO_3_^−^ was produced during the test process using the annealed photocatalyst at 300°C for 30 min (Figure S3b). The dependence of photocatalytic performances on oxygen vacancy concentrations is shown in Figure S4, and ultraviolet LED with high power was used to produce more NO_3_^−^ for activity comparison. The yields of NH_4_^+^ and NO_3_^−^ reduce as the annealing time (1.5, 3, and 30 min) increases at 300°C under a 300 W xenon lamp and 5 W of 370 nm LED illumination, respectively. The standard curves for quantifying ammonia and nitrate concentrations were calibrated using ion chromatography, which is a precise measurement for most anions and cations. The correlation coefficient values are 0.9993 and 0.9974 for ammonia and nitrate calibration curves (Figure S5), respectively. The peak signal from the lowest calibrated concentration (0.05 ppm) is very sharp and clean (Figures S5e and S5f), indicating that the instrument is capable of reliably measuring low concentrations at this level. Though some measured values for ammonia or nitrate listed in Table S1 are below the lowest calibrated concentration, the linear relationship between the peak area and the concentration of ammonia or nitrate should persist down to the origin. In other words, the calibrations in Figure S5 are acceptable to quantify the amount of ammonia and nitrate produced in the photocatalytic N_2_ fixation of this work.

To evaluate the photocatalytic stability, the suspension of the as-synthesized W_18_O_49_ nanowires was irradiated using a 300 W xenon lamp and tested for ten 12-hour cycles. After each cycle, the catalyst was carefully cleaned by filtration using a copious amount of distilled water to wash off the dissolved NH_4_^+^ and nitrate products. Even if a layer of NH_4_^+^ might strongly adsorb on the surface of the W_18_O_49_ catalyst and even if they are carried over to the next cycle, it is expected that the adsorbed NH_4_^+^ cannot easily desorb and only the NH_4_^+^ in the bulk solution can be extracted and measured. It is found that the as-synthesized W_18_O_49_ nanowires are relatively stable during the cycle test (Figure S3c). The UV-vis absorption spectrum of the sample after one cycle test (12 h) exhibits lower tail absorption intensity compared with that of the as-synthesized W_18_O_49_ nanowires (Figure S6), indicating that the concentration of oxygen vacancies decreases slightly. It can be speculated that the number of oxygen vacancies gradually decreased after each cycle according to the reduced photocatalytic activities as shown in Figure S3c. The total turnover number is greater than 114.7% after ten cycles, which confirms the photocatalytic reaction for N_2_ fixation.

In order to understand the wavelength-dependent catalytic process for N_2_ fixation, the as-synthesized photocatalyst suspension was irradiated under LED lights of different wavelengths: 365, 384.3, 400, 427, 468.4, 498, 515, 590, 620, 730, and 850 nm. It was found that the as-synthesized W_18_O_49_ nanowires can photocatalytically fix N_2_ to NH_4_^+^ and NO_3_^−^ at wavelengths from ultraviolet up to 730 nm (Figure 2(b) and Figure S7 and Table S1). Although there have been some visible light-sensitive photofixation catalysts reported, our photocatalyst appears to be the one with the widest absorption range so far [14, 34, 35]. Figure 2(c) demonstrates the molar percentage ratio of NH_4_^+^ and NO_3_^−^ to the total production. The ratio of NH_4_^+^ gradually decreases from 365 nm to 427 nm and increases from 427 nm to 730 nm with a high NO_3_^−^ yield (35~40%) during 427~515 nm; the reason will be discussed herein below.

The wavelength-dependent quantum efficiencies (QE) can also be evaluated based on the amount of photofixation products under LED light illumination using the equations [1, 2] in the experimental part. The calculated QE values are closely related to the wavelength and the light absorption ability. The trend of the photon-to-product efficiency *vs.* wavelength follows an inverted volcano shape in the range from 365 to 498 nm (Figure 2(d)). In this range, the highest and lowest efficiencies are 9% at the wavelength of 365 nm and 5% at 427 nm, respectively, where the light absorption ability is weakest. On the other hand, the absorption edge of the as-synthesized W_18_O_49_ nanowires is about 428 nm because the band gap was close to the incident energy of the light source (Figure S8a). The absorption in longer wavelengths must be caused by the defect levels (DLs). According to the density of states (DOS) calculation, there are some defect levels located below the conduction band in the presence of oxygen vacancies in W_18_O_49_ (Figures S8b and S8c). The DLs explain why the as-synthesized W_18_O_49_ nanowires exhibit tail absorption in the UV-vis absorption spectrum transformed from the Kubelka-Munk formula (Figures S6 and S8a). Since the density of defects is expected to decrease after annealing, the absorption in the tail range for the annealed sample for 30 min is lower (Figure S8a), suggesting that the photocatalytic activity above 428 nm wavelength originates from the DLs. Photon-to-product efficiency gradually decreases in the wavelength range from 498 nm to 730 nm, possibly due to the relatively lower energy of the photoexcited carriers. This trend is proven by the real production of NH_4_^+^ and NO_3_^−^ under different wavelength LED irradiation with approximate light intensity (Figure S7).

In order to determine the source of the oxygen element in the nitrate product, appropriate H_2_^18^O in normal distilled water was used as the reagent. After 5 h of xenon lamp irradiation, the ^18^O isotope was quantified by the denitrification method using Delta V-Precon (Thermo Fisher Scientific, Germany, with the detailed measurement method described in Supplementary Materials) to be about 0.20% in NO_3_^−^. The measured peak position, peak area, and atomic percentage are shown in Figure S9 and Table S2, and all the calculation values were based on the equipped software on the test instrument. For additional confirmation, control experiments were carried out to prove the photocatalytic nitrogen fixation ability and exclude possible interference from any contaminants. Firstly, neither ammonia nor nitrate can be detected in pure water (100 mL) with the as-synthesized W_18_O_49_ photocatalyst (0.05 g) and Ar gas bubble under 300 W xenon lamp irradiation at 25°C (Figure S10a). This result demonstrates that the nitrogen element in the ammonia and nitrate is from the N_2_ flow through photocatalytic fixation. Secondly, ammonia and nitrate are also undetectable in pure water (100 mL) with the as-synthesized W_18_O_49_ photocatalyst (0.05 g) and N_2_ gas bubble without irradiation at 25°C (Figure S10b). Then, light illumination is required for N_2_ fixation and the fixation products are not from environmental contaminations. These results unequivocally confirm that the photocatalytic reaction of N_2_ fixation to ammonia and nitrate indeed happens in this process, and the oxygen element in nitrate originates from water.

**Figure 3:**
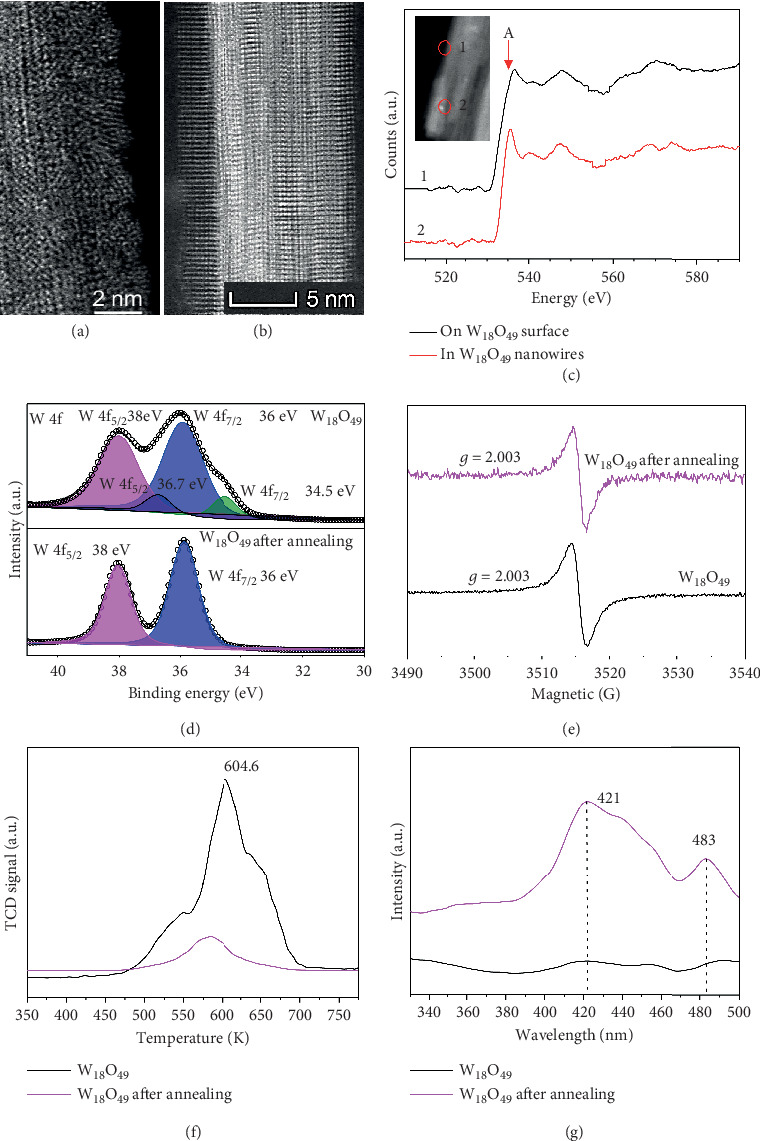
Analysis of surface defects in the as-synthesized and annealed W_18_O_49_ nanowires for 30 min at 300°C. (a) The surface of the W_18_O_49_ nanowires; the marked dash line is the polycrystalline and amorphous regions. (b) The surface of the annealed W_18_O_49_ nanowires. (c) EELS spectra of W_18_O_49_ nanowires. (d) High-resolution XPS W 4f of the original and annealed W_18_O_49_ nanowires. (e) The EPR spectra of the original and annealed W_18_O_49_ nanowires. (f) N_2_-TPD profiles of the original and annealed W_18_O_49_ nanowires. (g) The PL spectra of the original and annealed W_18_O_49_ nanowires.

The surface defects of the as-synthesized and annealed W_18_O_49_ nanowires are analyzed using atomic scale HAADF Z-contrast images. Lattice distortion, polycrystalline, and amorphous regions are revealed on the surface of the as-synthesized W_18_O_49_ nanowires (Figure 3(a)). However, all the surface irregularities disappear and the surface becomes smooth after annealing (Figure 3(b) and Figure S11). The electron energy loss spectroscopy (EELS) edges, which are sensitive to the unoccupied local density of states, provide useful information of local oxidation states and coordination chemistry of the W_18_O_49_ nanowires [36]. The W and O EELS results of the as-synthesized W_18_O_49_ nanowires are plotted in Figure 3(c), where peak A corresponds to the vacant density of states in the hybridized O 2p and W 5d orbitals. Therefore, the intensity of peak A is closely related to the oxidation state of W. As shown in the spectra collected inside the bulk and near the surface of the as-synthesized W_18_O_49_ nanowires, there is a significant drop in the intensity of peak A, which indicates a decrease in the valence state of W at the surface of W_18_O_49_ nanowires. For the W_18_O_49_ nanowires after annealing for 30 min, no visible change was observed in the EELS signals from the surface to the inside.

The chemical composition and the valence states of the as-synthesized and annealed W_18_O_49_ nanowires were examined with an X-ray photoelectron spectrometer (XPS). In the full range of XPS spectra, peaks at binding energies corresponding to O and W elements are clearly observed, and no impurities other than carbon are observed in the spectra (Figure S12a). The W 4f core-level spectrum of the as-synthesized sample could be fitted into two doublets with two different oxidation states. The main peaks of W 4f5/2 at 38 eV and W 4f7/2 at 36 eV are attributed to the W^6+^ oxidation state. The second doublet with a lower binding energy at 34.5 eV and 36.7 eV arises from W 4f5/2 and W 4f7/2 core levels of the W^5+^ oxidation state. These binding energies belong to the typical oxidation states found in W_18_O_49_ nanowires as reported previously [24, 37–39]. The above results further confirm that the as-synthesized catalyst is W_18_O_49_ rather than WO_3_. However, the peaks attributed to the W^5+^ oxidation state disappear after annealing, indicating that the concentration of surface oxygen vacancies decreases significantly after annealing, which is consistent with the EELS (Figure 2(c)). The O 1s high-resolution XPS spectra of W_18_O_49_ can be described as the deconvolution into two peaks by the Gaussian distribution (Figures S12b and S12c), where the one at 530.7 and 530.8 eV can generally be assigned to the bridging oxygen on the W_18_O_49_ surface, while the peak at 531.5 eV is attributed to O-H in the oxygen defects [40–42].

Electron paramagnetic resonance (EPR) spectroscopy and temperature-programmed desorption of N_2_ (N_2_-TPD) were used to investigate the oxygen vacancies and N_2_ adsorption on the surface oxygen vacancies. In Figure 3(e), a signal exists at around *g* = 2.003 caused by oxygen vacancies in the as-synthesized and annealed sample for 30 min [12]. Thus, there are still some oxygen vacancies in the annealed sample. In Figure 3(f), a single desorption peak of N_2_ begins at 450 K and centers at 604.6 K, of which the positions suggest chemisorption of nitrogen on the surface. A first-order process was indicated with the N_2_ peak unchanged with coverage for adsorption at 545 K. N_2_ was the major desorption product, with a peak at 650 K on Ru/Al_2_O_3_ [43–46]. Since the weights of samples were identical to these two TPD runs, the difference in peak areas can be used to compare the amount of N_2_ desorbed. For the annealed sample, the N_2_ TCD signal of the annealed W_18_O_49_ dramatically decreases in comparison with that of the as-synthesized sample, suggesting that there was a significant drop in the population of surface oxygen vacancies after annealing. In combination with the EPR and TCD results, it can be concluded that the surface oxygen vacancies are reduced but still expected to exist inside the annealed sample.

The PL emission spectra of the as-synthesized and annealed W_18_O_49_ nanowires for 30 min were examined in the wavelength range of 330-500 nm with an excitation wavelength of 280 nm at room temperature (Figure 3(g)). The blue emission peak at 421 nm is attributed to the oxygen vacancies in tungsten oxide nanowires and nanorods [47, 48], and the green emission peak centered at 483 nm is usually related to the intrinsic defect structures reduced particularly from oxygen deficiency [49]. The increased PL intensity demonstrates that more photogenerated electron-hole recombination occurs in the annealed sample. The PL result reveals that oxygen vacancies assist the separation of the photoexcited carriers, possibly by functioning as trapping sites.

The specific surface area is a key factor for the photocatalytic performance. The N_2_ adsorption and desorption isotherms of the as-synthesized and annealed W_18_O_49_ nanowires for 30 min were measured to evaluate their BET surface areas. The isotherms of both samples are of classical type IV with a hysteresis loop (Figure S13). The BET surface areas of the original W_18_O_49_ and annealed W_18_O_49_ were calculated to be 437.1 and 93.2 m^2^ g^−1^, respectively. Apparently, the annealing process resulted in a significant reduction in the surface area, most likely due to the change in the shape of the nanowires with the reconstruction of the rough surface. Although the specific surface area of the annealed sample was below one-fourth that of the as-synthesized sample, the photocatalytic activity turned out to be below 1/30 of the as-synthesized sample (Figure S3b). Therefore, the reduction in the specific surface area of the annealed sample alone cannot account for the significant decrease in the photocatalytic performance. It is the surface oxygen vacancy that primarily determines the performance of photofixation of N_2_.

**Figure 4:**
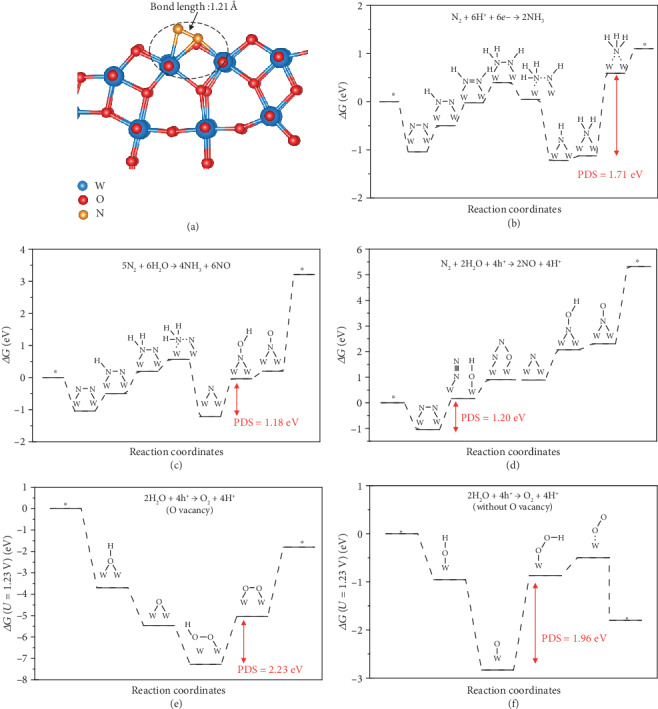
Theoretical calculation results. (a) The optimized structure of N_2_ adsorption configuration on the W_18_O_49_ (001) facet with one oxygen vacancy. (b–d) Free energy changes of nitrogen fixation reactions against the reaction coordinate on the W_18_O_49_ (001) facet with one oxygen vacancy. (b) The pathway for nitrogen reduction reaction to the NH_3_ product. (c) The pathway for nitrogen disproportionation into NH_3_ and NO products. (d) The pathway for NOR to the NO product. The free energy changes of OER at equilibrium potential *U* = 1.23 V on the W_18_O_49_ (001) facet (e) with and (f) without an oxygen vacancy.

In order to better understand the mechanisms for nitrogen fixation on the W_18_O_49_ nanowire from a microscopic point of view, density function theory calculation was carried out. The unit cell of W_18_O_49_ was optimized (Figure S14), and the relaxed lattice parameters (*a* = 18.50 Å, *b* = 3.82 Å, *c* = 14.19 Å, and *β* = 115.62°) are consistent with pervious work [14]. The (001) surface was modelled with a 1 × 2 × 1 supercell to investigate the N_2_ fixation process. We found that the N_2_ molecule cannot be chemically adsorbed on a perfect W_18_O_49_ surface (Figure S15), in which case the bond length of the adsorbed N_2_ is nearly identical to that of the gaseous phase. When an oxygen vacancy was introduced (Figure 4(a)), however, the Bader charge analysis showed that 0.85 *e*^−^ is localized on each of the two W atoms around the oxygen vacancy. Therefore, both N atoms of N_2_ could form strong bonds with the W atoms around the oxygen vacancy, and the N-N bond length is significantly stretched from 1.11 Å to 1.21 Å with adsorption energy of -1.70 eV, indicating the activation of the N_2_ triple bond. The charge difference analysis with Bader charge analysis was performed to analyze the charge of N_2_ adsorption configuration (Figure S16 and Table S3). It is found that the adsorbed N_2_ gain 0.74 *e*^−^ and the charges accumulate in the area between the bonded N and W atoms, while a charge depletion region is created between both N atoms, indicating that the N_2_ triple bond is weakened, thus facilitating the following nitrogen fixation reactions [50]. Free energy profiles toward different products were calculated under pH = 7 (Figures 4(b)–4(f)), and the optimized geometries for the reaction intermediates are presented in Figures S17–S22. For NRR, the most energetically favorable pathway is shown in Figure 4(b). In this process, the potential-determining step (PDS) is the last hydrogenation step (∗NH_2_-∗NH_3_) and the corresponding energy (Δ*G*_PDS_) is 1.71 eV. In NOR, N_2_ can be oxidized to produce two NO molecules (Figure 4(d)) with Δ*G*_PDS_ of 1.20 eV. Intriguingly, it is found that N_2_ can also disproportionate at a single oxygen vacancy, forming NH_3_ and NO successively (Figure 4(c)). The PDS is that the hydroxyl attacks the ∗N intermediate to form the ∗NOH intermediate and Δ*G*_PDS_ is 1.18 eV, demonstrating that such disproportionation reaction probably prevails in the N_2_ fixation process. The pathways for the OER on the W_18_O_49_ (001) with an oxygen vacancy and the pristine W_18_O_49_ (001) are conceived from studies of OER on other metal oxides and depicted in Figures 4(e) and 4(f). According to the calculated Δ*G*_PDS_ for both pathways, OER appears to be more feasible on a facet without an oxygen vacancy. The formation of H_2_O_2_ was also examined (Figure S23), and it turned out that H_2_O_2_ can hardly take part in the reactions of our system.

**Figure 5:**
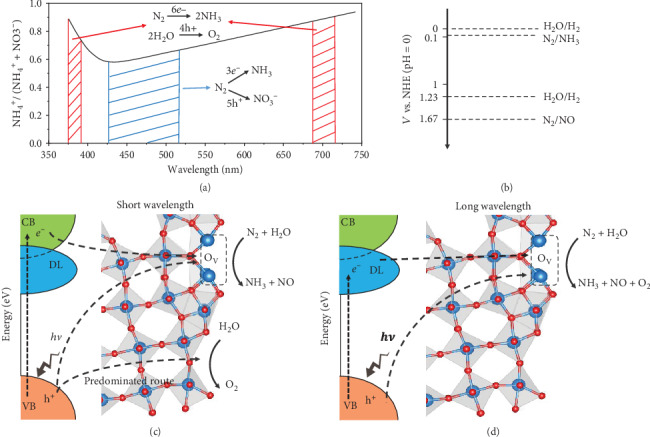
(a) Sketch diagram of the molar percent ratio of NH_4_^+^ to the total products. (b) Thermodynamic conditions of water reduction and oxidation to H_2_ and O_2_ and N_2_ reduction and oxidation to NH_3_ and NO (NHE: normal hydrogen electrode, pH = 0). Proposed mechanisms of photocatalytic reactions during N_2_ fixation in (c) short and (d) long wavelength ranges.

Although many factors may play a role in the entire photocatalytic process of N_2_ fixation on the W_18_O_49_ nanowires, we believe that the oxygen vacancies on the nanowire surface are essential in promoting the chemical adsorption of N_2_ molecules and providing catalytic-active sites for both ammonia and nitrate formations. Here, we propose the wavelength-controlled mechanism of photocatalytic N_2_ fixation on W_18_O_49_ nanowires (Figure 5). Under the whole range light irradiation from 365 to 730 nm used in this work, the photoexcited electrons transfer to the surface oxygen vacancies and reduce the chemisorbed N_2_ molecule to NH_3_ (Figure 5(a)). However, in the short wavelength range from 365 nm to around 427 nm, intrinsic absorption of light is valid and the photoexcited holes are generated in the bulk and near the surfaces. In this case, the highly mobile holes are delocalized over the surface regions and can reach the W sites either with or without a nearby oxygen vacancy. Owing to the favorable OER at the W sites without a nearby oxygen vacancy (Figures 4(e) and 4(f)), only a small portion of photoexcited holes are injected to the oxygen vacancies where NOR takes place. The OER takes more advantage under the shorter wavelength light irradiation (Figure 5(c)), which corresponds to the increase in the ratio of NO_3_^−^ at wavelength from 365 nm to around 427 nm (Figure 2(c)). In the long wavelength from around 427 nm to 730 nm (Figure 5(d)), intrinsic absorption is not available; hence, all the reactions are most likely to occur on oxygen vacancies due to light absorption by DLs. Since the equilibrium potential of the N_2_/NO redox couple is 0.44 eV higher than that of the H_2_O/O_2_ redox couple (Figure 5(b)), OER will be more thermodynamically favorable than NOR at longer wavelength. As a result, the ratio of NO_3_^−^ decreases in this range of wavelength (Figure 2(c)). It is worth mentioning that for wavelength from 427 nm to 515 nm, the ratio between the produced NH_4_^+^ and NO_3_^−^ is close to 5 : 3. Given that the valence changes of N from N_2_ to NH_4_^+^ and NO_3_^−^ are -3 and 5, respectively, we can deduce that nearly all the O_2_ molecules produced by OER are consumed by the oxidation of NO to NO_3_^−^, i.e., through Reaction (4). Under this condition, O_2_ should be regarded as a reaction intermediate rather than a reaction product, and all the photoexcited holes that participate in reactions will take part in the oxidation of N_2_. In other words, the photogenerated carriers are most efficiently utilized in this wavelength range.

## 3. Conclusions

In summary, we have developed oxygen vacancy-rich W_18_O_49_ ultrathin nanowires as an excellent photocatalyst for N_2_ fixation into ammonia and nitrate. Our investigation revealed that the oxygen vacancies promote the light absorption from the visible to the NIR region, improve the separation ability of the photoexcited electrons and holes, and also serve as the active sites for N_2_ chemisorption and the bridging between the photogenerated carriers from the catalyst to the N_2_ molecules. The total quantum efficiency can reach 9% at the irradiation wavelength of 365 nm. Theoretical results show that the oxygen vacancies are the catalytic sites for the formation of both ammonia and nitrate. Interestingly, the molar percentage ratio of NH_4_^+^ to the total production (NH_4_^+^ and NO_3_^−^) shows a gradual decrease from 365 nm to 427 nm, followed by an increase from 427 nm to 730 nm. This trend can be rationalized as follows: in the short wavelength range, the energetically favorable OER predominates at the W sites without a nearby oxygen vacancy due to the intrinsic absorption of the catalyst and the delocalized nature of the photoexcited holes; in long wavelength ranges, NOR becomes more energetically challenging as compared with OER at oxygen vacancies according to the equilibrium potential for both reactions. The photogenerated carriers are most efficiently utilized in the wavelength range from ~427 nm to ~515 nm. This work presents a new insight into the role of oxygen vacancies in the wavelength-dependent photocatalytic nitrogen fixation and demonstrates the underlying mechanisms that could guide the design of future photocatalysts of higher efficiencies.

## Supplementary Materials

Supplementary Materials Preparation of W_18_O_49_ nanowires. Material characterizations. Photocatalytic activity evaluations. Isotope test. Figure S1: the XRD patterns of the original and annealed W_18_O_49_ nanowires. Figure S2: (a) SEM, (b) TEM, and (c) HRTEM images of the annealed W_18_O_49_ nanowires. Figure S3: the photocatalytic NH_4_^+^ and NO_3_^−^ production ability of the (a) as-synthesized W_18_O_49_ nanowires and (b) annealed W_18_O_49_ nanowires for 30 min under a xenon lamp. (c) Stability of W_18_O_49_ nanowires for N_2_ reduction under a xenon lamp (light source: 300 W xenon lamp; 25°C). Figure S4: (a) NH_4_^+^ yield under 300 W of xenon lamp illumination and (b) NO_3_^−^ yield under 5 W of 370 nm LED illumination for photocatalytic N_2_ fixation of the as-synthesized W_18_O_49_ nanowires (0.05 g) and the sample annealed for different times at 300°C. Figure S5: standard curve of (a) nitrate and (b) ammonia with high-performance ion chromatography. (c) Measurement curves of NH_4_^+^ from 0.05 ppm to 1 ppm tested by ion chromatography. (d) Measurement curves of NO_3_^−^ and NO_2_^−^ from 0.05 ppm to 1 ppm tested by ion chromatography. (e) The enlarged curves of (c). (f) The enlarged curves of (d). Figure S6: the UV-vis absorption spectra of the as-synthesized W_18_O_49_ nanowires and the as-synthesized sample after one cycle test of photocatalytic N_2_ fixation for 12 h. Figure S7: the photocatalytic NH_4_^+^ and NO_3_^−^ production ability of the as-synthesized W_18_O_49_ nanowires under different wavelength LED irradiation: (a) 384.3 nm; (b) 400 nm; (c) 427 nm; (d) 468.4 nm; (e) 498 nm; (f) 515 nm; (g) 590 nm; (h) 620 nm; (i) 850 nm. Figure S8: (a) the UV/vis absorption spectra of W_18_O_49_ nanowires and annealed W_18_O_49_ nanowires. (b) Density of states for W_18_O_49_. (c) Density of states for W_18_O_49_ with an oxygen vacancy. Figure S9: the typical measurement peaks of different N_2_O. Figure S10: (a) the ammonia and nitrate concentration curves with the as-synthesized W_18_O_49_ photocatalyst (0.05 g) and Ar gas bubble in water (100 mL) under 300 W of xenon lamp irradiation (25°C). (b) The ammonia and nitrate concentration curves with the as-synthesized W_18_O_49_ (0.05 g) photocatalyst and N_2_ gas bubble in water (100 mL) without irradiation (25°C). Figure S11: the HRTEM image of W_18_O_49_ nanowires after annealing. Figure S12: (a) XPS full-range spectra of the original and annealed W_18_O_49_ nanowires for 30 min. High-resolution XPS O 1s of (b) W_18_O_49_ nanowires. (c) Annealed W_18_O_49_ nanowires for 30 min. Figure S13: N_2_ adsorption/desorption isotherms at 77 K of W_18_O_49_ nanowires and annealed W_18_O_49_ nanowires. Figure S14: the top view, side view, and lattice parameters of the bulk unit cell of W_18_O_49_. Figure S15: the adsorption configuration of the N_2_ molecule on the perfect W_18_O_49_ (001) facet without an oxygen vacancy. Figure S16: the different oxygen vacancies on the W_18_O_49_ nanowires. Figure S17: the charge difference analysis of the N_2_ adsorption configuration on the W_18_O_49_ (001) facet with one oxygen vacancy; blue represents charge accumulation while red indicates charge depletion. Figure S18: the optimized geometries for reaction intermediates during the nitrogen fixation process to the NH_3_ product over W_18_O_49_ (001) with one oxygen vacancy. Figure S19: the optimized geometries for reaction intermediates during the nitrogen fixation process to NH_3_ and NO products over W_18_O_49_ (001) with one oxygen vacancy. Figure S20: the optimized geometries for reaction intermediates during the nitrogen fixation process to the NO product over W_18_O_49_ (001) with one oxygen vacancy. Figure S21: the optimized geometries for reaction intermediates during the OER process over W_18_O_49_ (001) with one oxygen vacancy. Figure S22: the optimized geometries for reaction intermediates during the OER process over the pristine W_18_O_49_ (001). Figure S23: formation of H_2_O_2_ on the W_18_O_49_ (001) facet (a) with and (b) without one O vacancy and (c) the 2∗OH and ∗O_2_H_2_ intermediates on the surface without an O vacancy. Table S1: the area of characteristic peak and corresponding ammonia and nitrate concentration. Table S2: the delta value of atomic percentage of ^18^O. Table S3: the formation energy of different oxygen vacancies.
