# Supplementary material for: Wavelength-Dependent Solar N2 Fixation into Ammonia and Nitrate in Pure Water
Source: Research (Wash D C). 2020 May 29;2020:3750314. doi: 10.34133/2020/3750314 (PMC7275971; doi:10.34133/2020/3750314)
Supplement: Supplementary Materials — Preparation of W18O49 nanowires. Material characterizations. Photocatalytic activity evaluations. Isotope test. Figure S1: the XRD patterns of the original and annealed W18O49 nanowires. Figure S2: (a) SEM, (b) TEM, and (c) HRTEM images of the annealed W18O49 nanowires. Figure S3: the photocatalytic NH4+ and NO3− production ability of the (a) as-synthesized W18O49 nanowires and (b) annealed W18O49 nanowires for 30 min under a xenon lamp. (c) Stability of W18O49 nanowires for N2 reduction under a xenon lamp (light source: 300 W xenon lamp; 25°C). Figure S4: (a) NH4+ yield under 300 W of xenon lamp illumination and (b) NO3− yield under 5 W of 370 nm LED illumination for photocatalytic N2 fixation of the as-synthesized W18O49 nanowires (0.05 g) and the sample annealed for different times at 300°C. Figure S5: standard curve of (a) nitrate and (b) ammonia with high-performance ion chromatography. (c) Measurement curves of NH4+ from 0.05 ppm to 1 ppm tested by ion chromatography. (d) Measurement curves of NO3− and NO2− from 0.05 ppm to 1 ppm tested by ion chromatography. (e) The enlarged curves of (c). (f) The enlarged curves of (d). Figure S6: the UV-vis absorption spectra of the as-synthesized W18O49 nanowires and the as-synthesized sample after one cycle test of photocatalytic N2 fixation for 12 h. Figure S7: the photocatalytic NH4+ and NO3− production ability of the as-synthesized W18O49 nanowires under different wavelength LED irradiation: (a) 384.3 nm; (b) 400 nm; (c) 427 nm; (d) 468.4 nm; (e) 498 nm; (f) 515 nm; (g) 590 nm; (h) 620 nm; (i) 850 nm. Figure S8: (a) the UV/vis absorption spectra of W18O49 nanowires and annealed W18O49 nanowires. (b) Density of states for W18O49. (c) Density of states for W18O49 with an oxygen vacancy. Figure S9: the typical measurement peaks of different N2O. Figure S10: (a) the ammonia and nitrate concentration curves with the as-synthesized W18O49 photocatalyst (0.05 g) and Ar gas bubble in water (100 mL) under 300 W of xenon lamp ir [file 3750314.f1.docx]

Supplementary Materials for

**Wavelength-dependent Solar N_2_ Fixation into Ammonia and Nitrate in Pure Water**

Wenju Ren^†^, Zongwei Mei^†^, Shisheng Zheng^†^, Shunning Li^†^, Yuanmin Zhu, Jiaxin Zheng, Yuan Lin, Haibiao Chen, Meng Gu, Feng Pan*

Correspondence to: [panfeng@pkusz.edu.cn](mailto:panfeng@pkusz.edu.cn)

**Preparation of W_18_O_49_ nanowires.** The monoclinic W_18_O_49_ nanowires were prepared using a solvothermal method. All the chemical reagents employed in this study were analytical grade from Aladdin and used without further purification. In a typical synthesis process, WCl_6_ was dissolved into 50 mL of absolute ethanol to form a 10 mM orange yellow solution. Then the solution was transferred into a Teflon-lined stainless-steel autoclave, and subsequently sealed and heated at 200 °C for 24 h. After the reaction, a blue flocculent precipitate was collected and washed with ethanol and water, followed by lyophilization. In order to reduce the oxygen vacancy concentration, the blue product was annealed at 300 °C in the air for 1.5, 3 or 30 minutes, then the samples were correspondingly labelled as W_18_O_49_-1.5, W_18_O_49_-3, and W_18_O_49_-30.

**Material characterizations.** X-ray diffraction data (XRD) were collected with a Bruker D8-Advance diffractometer (40 KV, 40 mA Cu Kα, λ = 1.5418 Å, resolution 0.02º). Scanning electron microscopy (SEM) images were taken on a ZEISS SUPRA®55 field emission SEM instruments and Transmission electron microscope (TEM) imaging and high resolution-transmission electron microscopy (HRTEM) were performed on a Double-Cs aberration-corrected Themis G2 microscope at 300 kV with a Quantum GIF detector. The absorption spectrum was measured using a Shimadzu UV-2450 spectrophotometer. Photoluminescence (PL) spectra were recorded on a Spex FL201 fluorescence spectrophotometer using 280 nm He-Cd laser as the excitation light. The X-ray photoelectron spectroscopy (XPS) analysis was carried out using a Thermo Scientific ESCALAB 250 Xi system. The Raman spectra were acquired using Horiba iHR320 with a 532.1 nm laser, and the accumulation time was 15 s. The surface area was calculated using the Brunauer-Emmett-Teller (BET) model by a Micromeritics ASAP 2020 HD88. The temperature-programmed desorption of N_2_ (N_2_-TPD) was tested with an Autosorb-iQ-C chemisorption analyzer (Quantachrome, USA) with the temperature from 0 to 773 K at a heating rate of 1.0 K/min.

**Photocatalytic activity evaluations.** The nitrogen photofixation performance was carried out in a double-walled quartz reactor. 0.05 g of photocatalyst was added into 100 mL of deionized water to form a suspension that was dispersed by ultrasonication for 10 min. And then the reactor was irradiated by a solar simulation light (AM 1.5G, 100 mW/cm^2^) or a 300 W Xe lamp (CEL-HXF300) after 0.5 h of nitrogen bubbling under mild stirring. The reactor temperature was held at 25 °C using a water thermostat. During the photocatalytic reaction process, 3 mL of the suspension was collected and immediately centrifuged to separate the dissolved products from the solid catalyst. The concentration of NH_4_^+^ and nitrate in the solution was measured by an ion chromatography (Metrohm Eco IC). And all the concentration values were based on the standard curve from different concentration of NH_4_^+^ and nitrate standards (Figure S5). For the isotope analysis, 1 mL of H_2_^18^O was added into 99 of mL water for photocatalytic reaction.

To evaluate the quantum efficiency (QE) of the as-synthesized W_18_O_49_ nanowires, LED lights with different wavelengths (365 nm, 384.3 nm, 400 nm, 416 nm, 468.4 nm, 498 nm, 515 nm, 590 nm, 620 nm, 730 nm, 730 nm, 850 nm) were used as the light source. The light intensity was tested by (Thorlabs, PM100D with S401C). The CQE values of ammonia and nitrate production were calculated based on the following equations (1) and (2).

$\mathrm{QE}_{ammonia}\left( \% \right)=100 \times\frac{number of molcules ammonia \times3}{number of incident photons}=\frac{100\times M\times N_{A}\times3}{P\times t\times\frac{\lambda}{hc}}$ (1)

$\mathrm{QE}_{nitrate}\left( \% \right)=100 \times\frac{number of molcules nitrate \times5}{number of incident photons}=\frac{100\times M\times N_{A}\times5}{P\times t\times\frac{\lambda}{hc}}$ (2)

where *M* represents the amount of ammonia or nitrate generated, NA is the Avogadro’s constant, *P* is the power of the incident light, $\lambda$ is light irradiation wavelength, *h* is Plank constant, *c* is the light speed, and *t* is the light irradiation time.

Turn over number (TON) is deﬁned as mole substrate reacted per mole catalyst. The TON was calculated followed equation (3).

$TON=100\times\frac{moles of ammonia and nitrate product}{moles of catalyst}=100\times\frac{(\frac{0.000246}{18}+\frac{0.00002}{62})}{\frac{0.05}{4093.12}}=114.7\%$(3)

**Isotope test.** The *δ*^18^O of NO_3_^-^ were determined by the “Bacterial denitrification method”, which allows for the simultaneous determination of *δ*^15^N and *δ*^18^O of N_2_O produced from the conversion of NO_3_^-^ by denitrifying bacteria (*Pseudomonas aureofaciens*, ATCC13985,USA), which naturally lack N_2_O-reductase activity. The *δ*^18^O analysis of the produced N_2_O was carried out using a trace gas preparation unit (Precon, Finnigan, Germany) coupled to an isotope ratio mass spectrometer (IRMS) (Delta V plus, Finnigan, Germany). The N_2_O sample is injected via an autosampler and H_2_O/CO_2_ is removed using scrubbers (Magnesium Perchlorate and Carbosorb, Merck KGaA). By cryogenic trapping and focusing, the N_2_O is compressed onto a capillary column (PoraPlot Q, 25 m, 0.32 mm id, 10 mm df, Agilent Technologies, US) at 35 °C and subsequently analyzed by IRMS. The *δ*^18^O were calibrated with USGS34 (27.8±0.4‰ for *δ*^18^O), IAEA N3(25.6±0.4‰ for *δ*^18^O) and USGS35 (56.8±0.3‰ for *δ*^18^O).

**Theoretical calculation.** All of the computations were performed by means of spin-polarized density functional theory with the Vienna ab initio simulation package (Vasp)(*1*). The generalized gradient approximation (GGA) in the Perdew-Burke-Ernzerhof (PBE) form and the projector augmented wave (PAW) potential were used(*2*, *3*). A 500 eV cut-off energy for the plan wave basis set was adopted. The coverage criteria of energy and force are 10^-5^ eV and 0.05 eV/Å, respectively. The (001) facet of W_18_O_49_ was chosen as the model surface. Supercells consisting of 1$\times$2$\times$1 unit cells, in which one oxygen atom was removed to create an oxygen vacancy, are used for geometrical optimization and static electronic structure calculation (Fig. S10 and Table. S1). At least 15Å layer of vacuum was added to avoid the interaction between two periodic units. During the optimization process, the bottom three layers of atoms were fixed. The K-point sampling of the Brillioun zone was done by 2$\times$5$\times$1 Monkhorst−Pack mesh. The Gibbs free energy profiles were calculated by the following equation(*4*) :

$\Delta G=\Delta E+\Delta E_{ZPE}-T\Delta S+\Delta G_{\mathrm{pH}}$ (4)

Where Δ*E* is the electronic energy difference directly calculated by DFT. Δ*E*_ZPE_ is the change in zero-point energy difference. T is the room temperature of 298.15K, and ΔS is the entropy change. Δ*G*_pH_ is the free energy correction with respect to pH of the solution, which can be determined using the following equation:

Δ*G*_pH_ =2.303$\times$K_b_T$\times$pH $\Delta G_{PH}=2.303\times K_{b}T\times\mathrm{pH}$  (5)

Where K_b_ is the Boltzmann constant and in order to simulate the real situation, the value of pH is assumed to be 7.


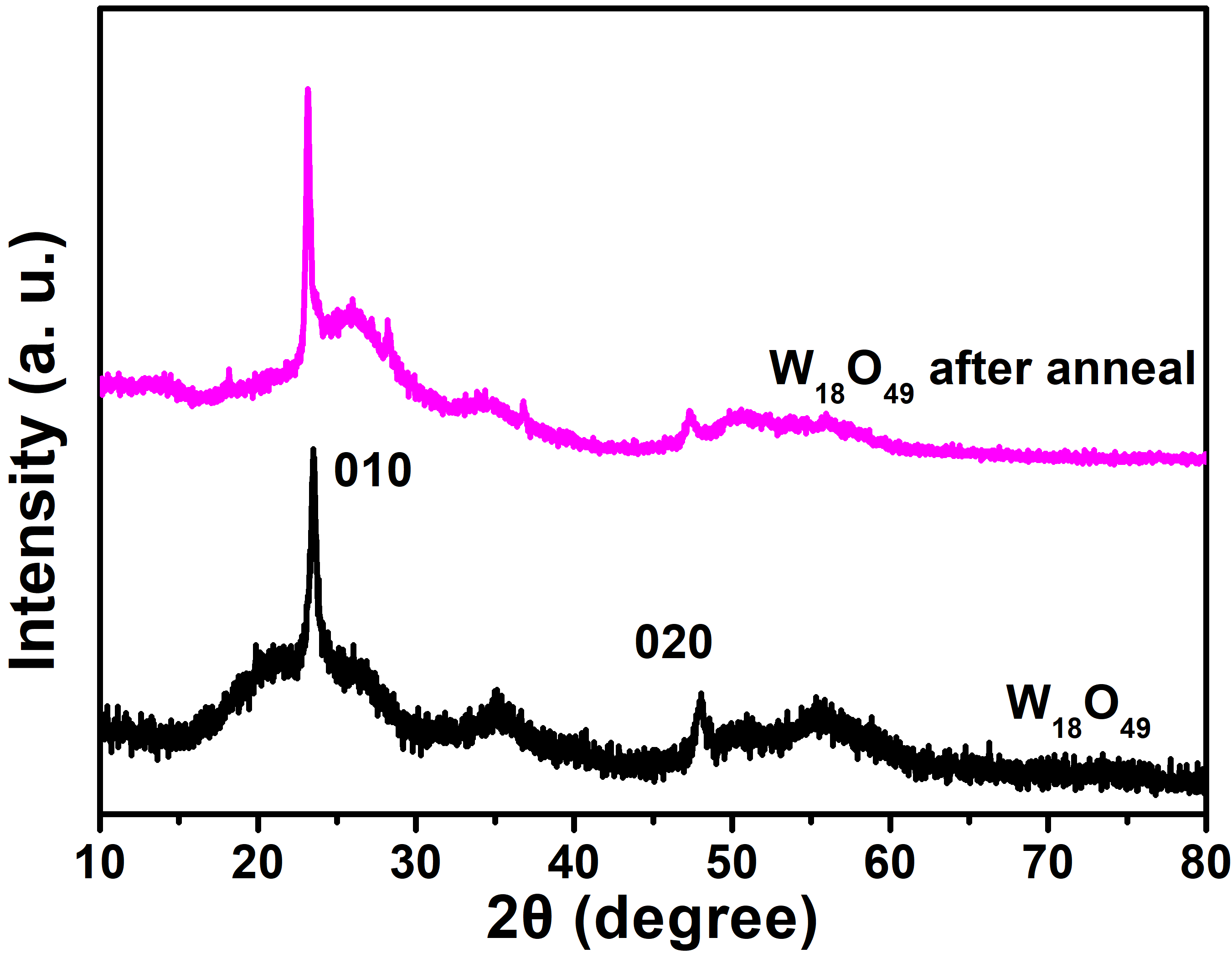


**Figure S1.** The XRD patterns of original and annealed W_18_O_49_ nanowires.


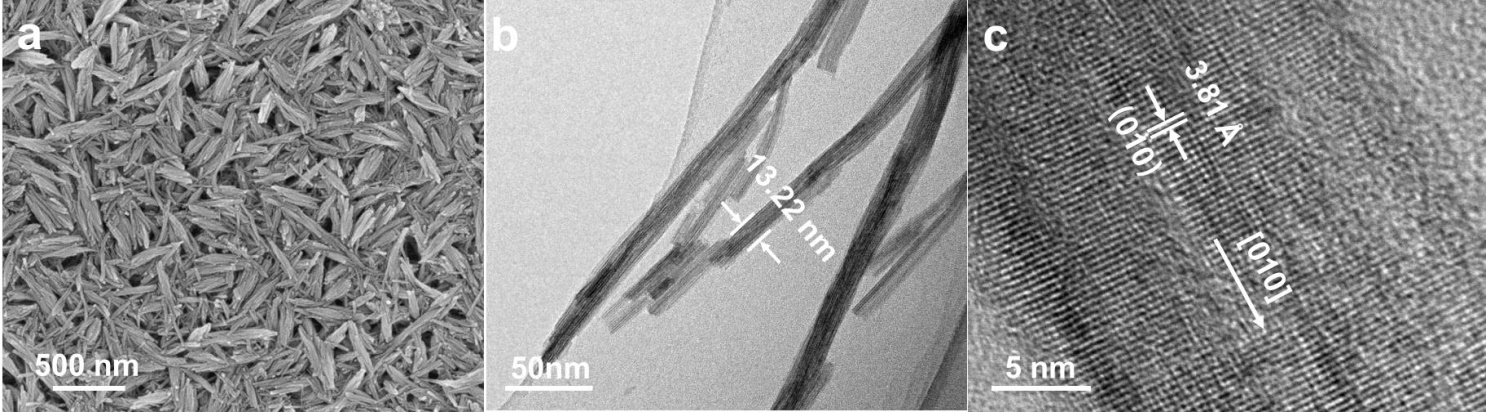


Figure S2. (a) SEM, **(**b**)** TEM and (c) HRTEM images of annealed W_18_O_49_ nanowires.


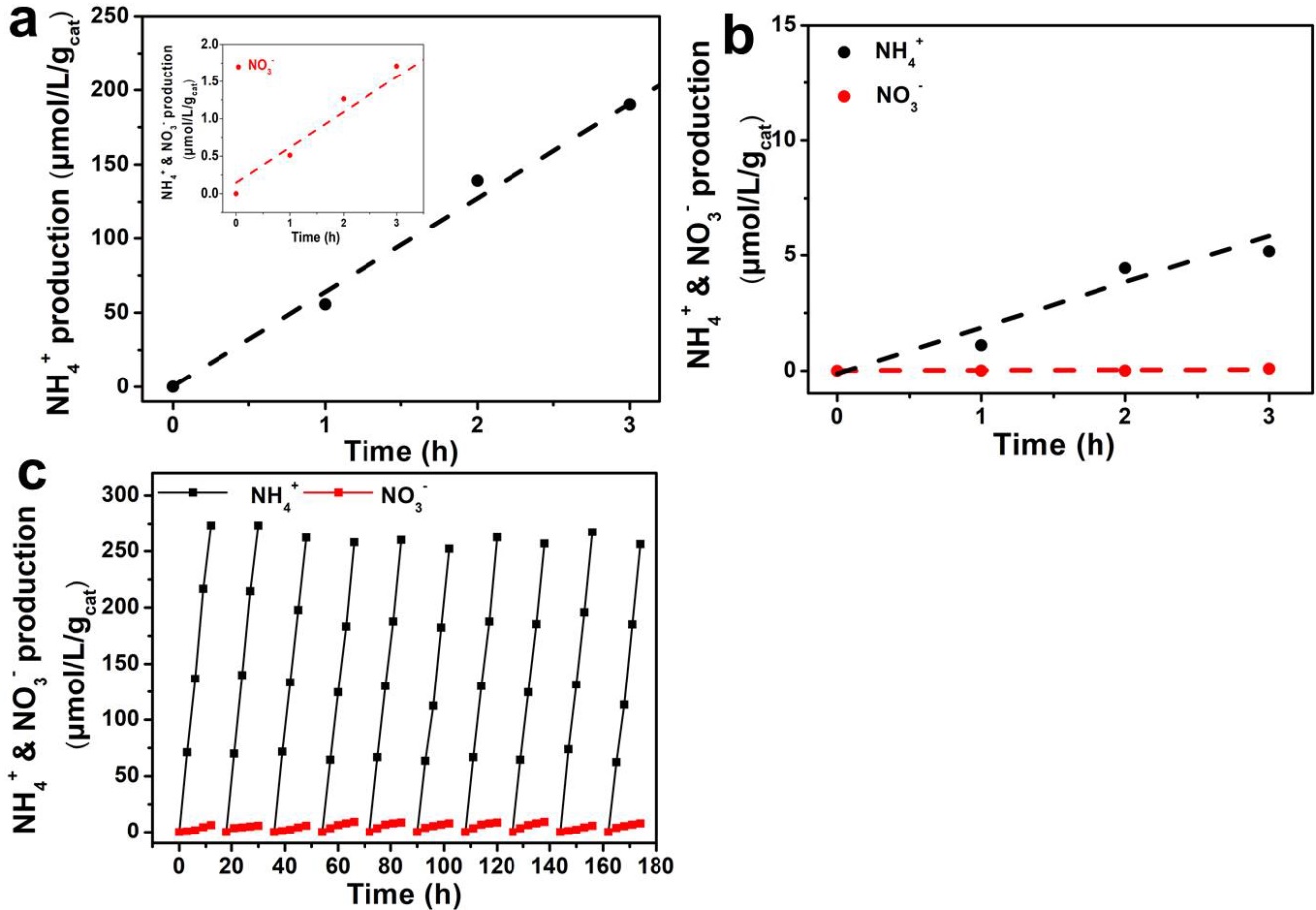


Figure S3. The photocatalytic NH_4_^+^ & NO_3_^-^ production ability of (a) as-synthesized W_18_O_49_ nanowires and (b) annealed W_18_O_49_ nanowires for 30 min under Xenon lamp. (c) Stability of W_18_O_49_ nanowires for N_2_ reduction under Xenon lamp (light source: 300 W xenon lamp; 25 °C).


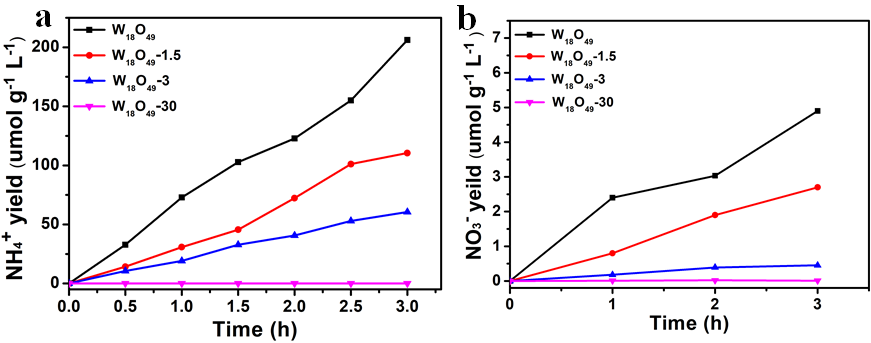


**Figure S4.** (a) NH_4_^+^ yield under 300 W of Xenon lamp illumination, and (b) NO_3_^-^ yield under 5 W of 370 nm LED illumination for photocatalytic N_2_ fixation of the as-synthesized W_18_O_49_ nanowires (0.05 g) and the sample annealed for different time at 300 °C.


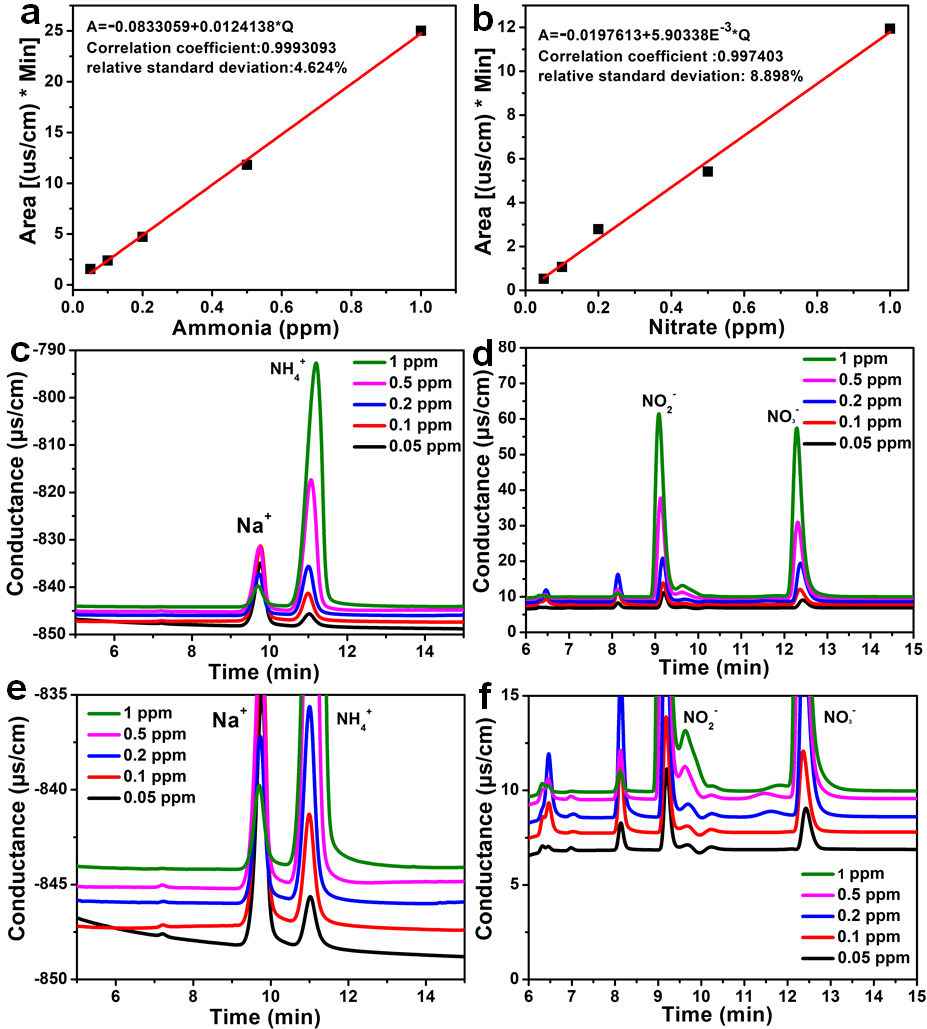


Figure S5. Standard curve of (a) nitrate and (b) ammonia with high-performance ion chromatography. (c) Measurement curves of NH_4_^+^ from 0.05 ppm to 1 ppm tested by the ion chromatography. (d) Measurement curves of NO_3_^-^ and NO_2_^-^ from 0.05 ppm to 1 ppm tested by the ion chromatography. (e) The enlarged curves of (c). (f) The enlarged curves of (d).


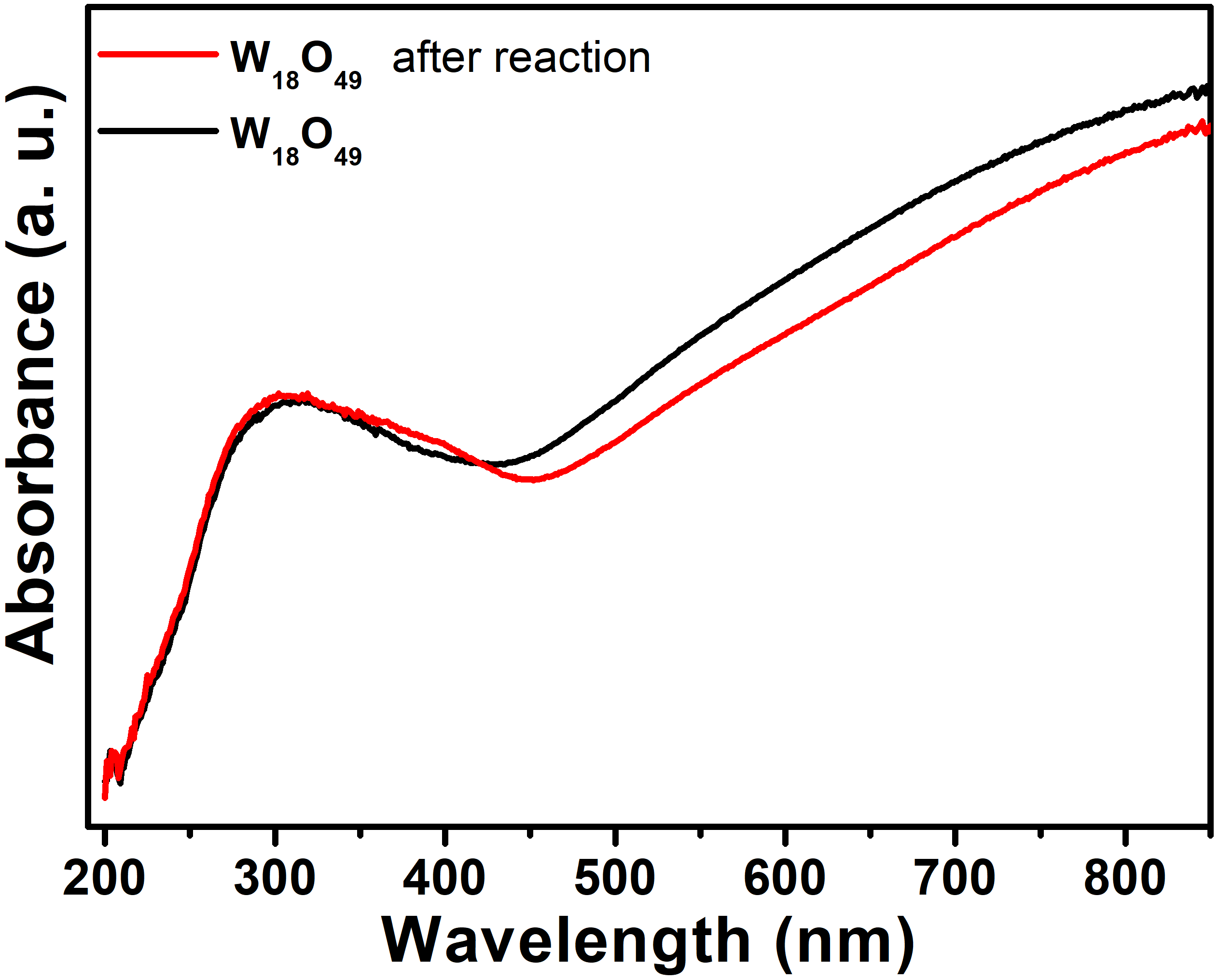


**Figure S6.** The UV-Vis absorption spectra of the as-synthesized W_18_O_49_ nanowires and the as-synthesized sample after one cycle test of photocatalytic N_2_ fixation for 12 h.

**
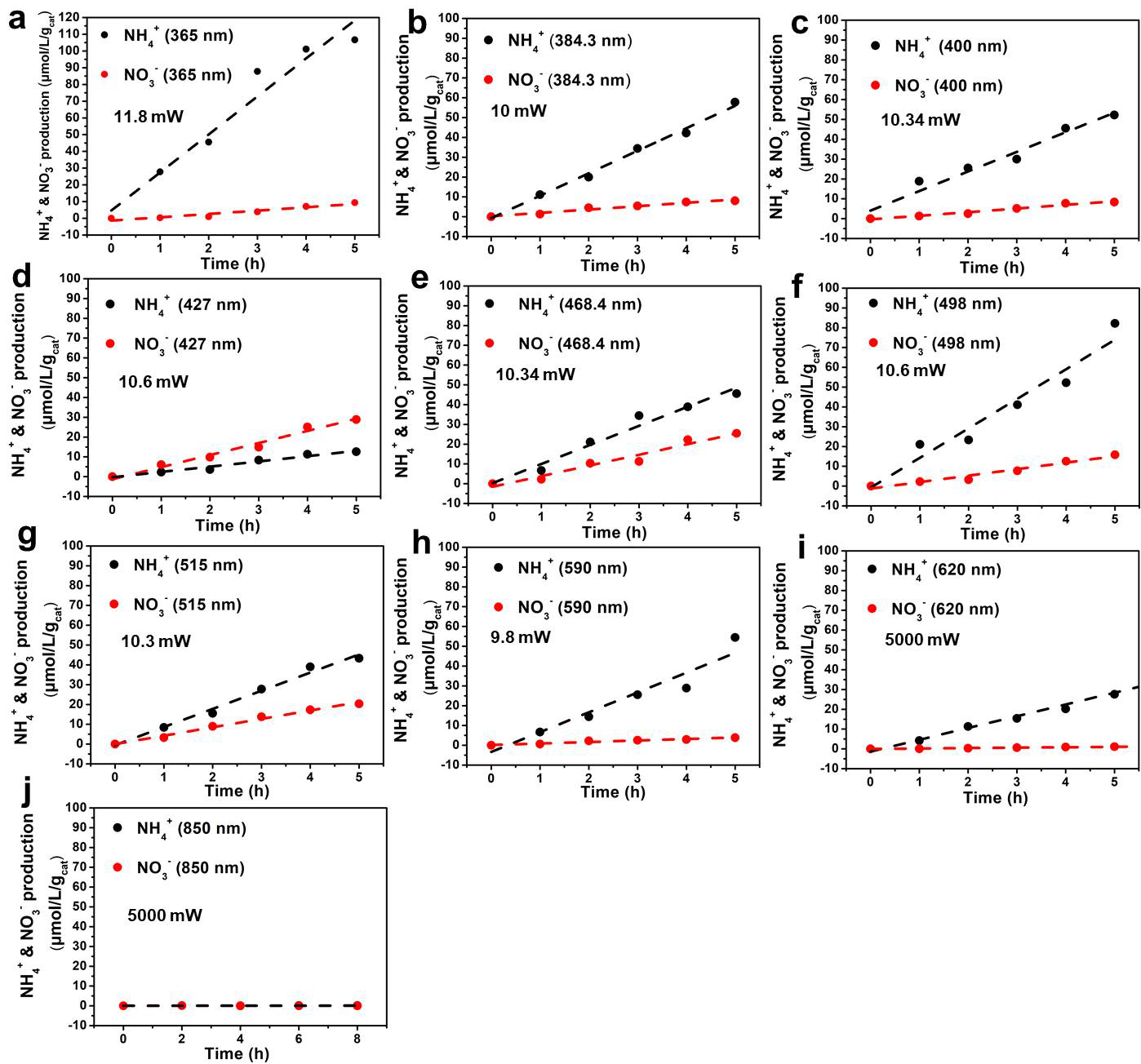
**

Figure S7. The photocatalytic NH_4_^+^ & NO_3_^-^ production ability of the as-synthesized W_18_O_49_ nanowires under different wavelength LED irradiation **(**a**)** 384.3 nm; **(**b**)** 400 nm; (c) 427 nm; (d) 468.4 nm; (e) 498 nm; (f) 515 nm; (g) 590 nm; (h) 620 nm; and (i) 850 nm.


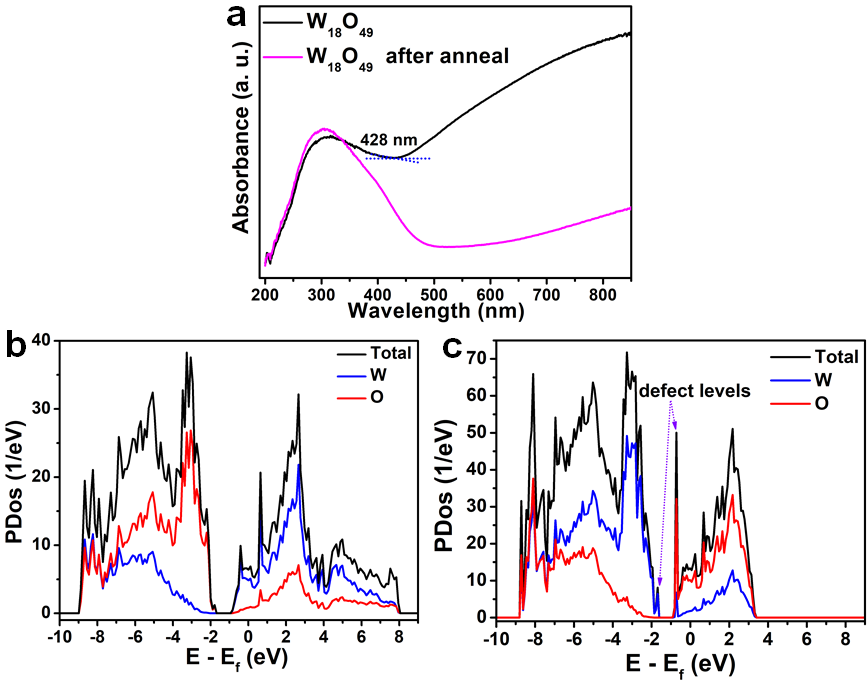


Figure S8. (a) The UV/Vis absorption spectra of W_18_O_49_ nanowires, annealed W_18_O_49_ nanowires. (b) Density of states for W_18_O_49_. (c) Density of states for W_18_O_49_ with oxygen vacancy.


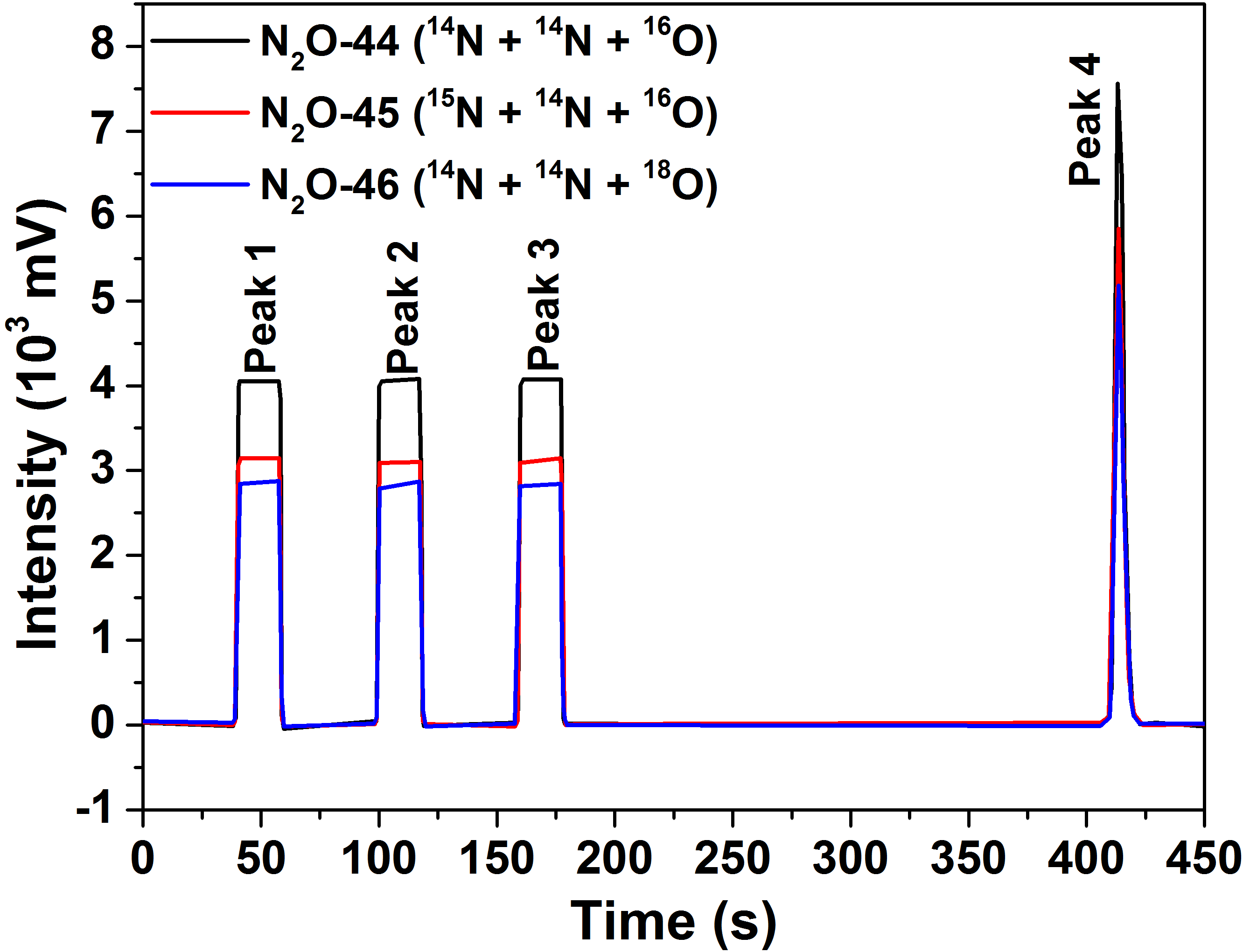


**Figure S9.** The typical measurement peaks of different N_2_O.


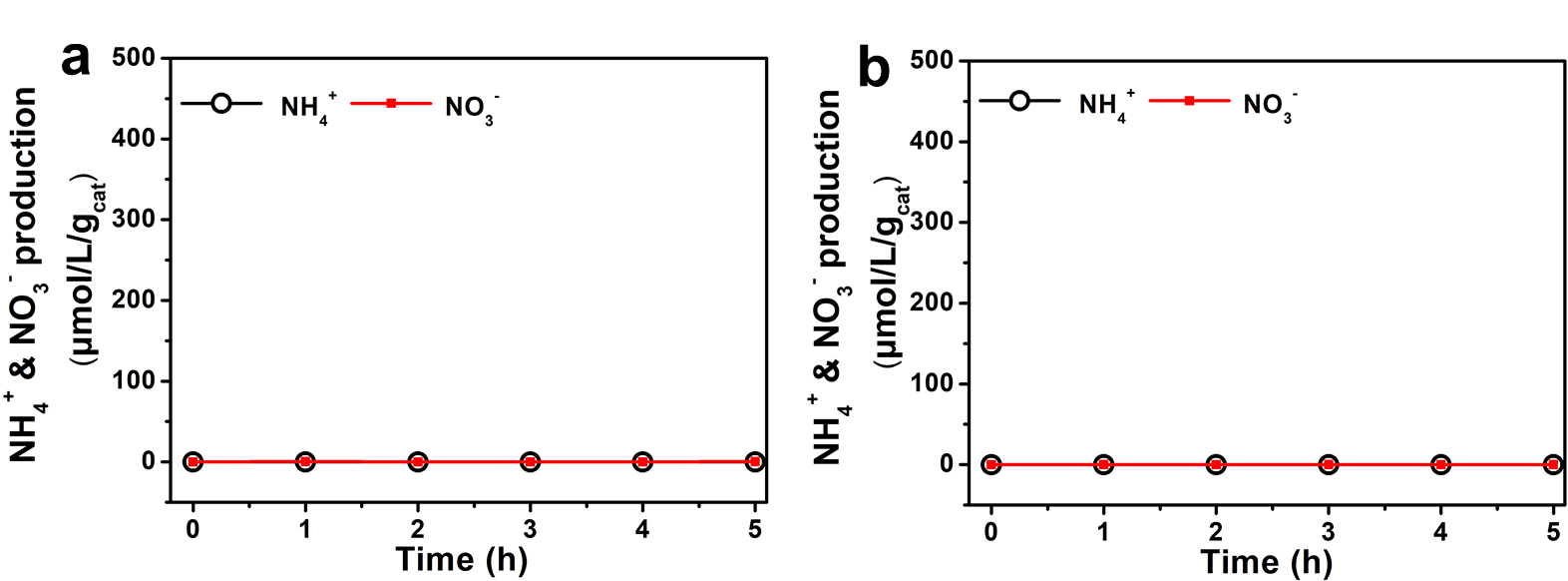


Figure S10. (a) The ammonia and nitrate concentration curves with the as-synthesized W_18_O_49_ photocatalyst (0.05 g) and Ar gas bubble in water (100 mL) under 300 W of xenon lamp irradiation (25 °C). (b) The ammonia and nitrate concentration curves with the as-synthesized W_18_O_49_ (0.05 g) photocatalyst and N_2_ gas bubble in water (100 mL) without irradiation (25 °C).


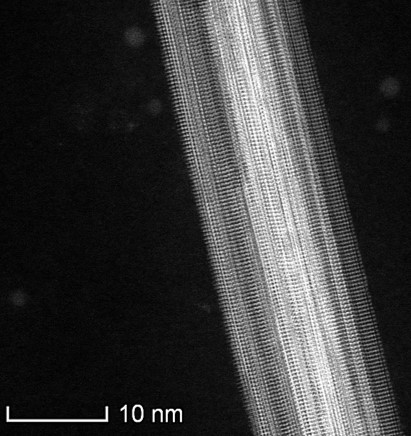


Figure S11. The HRTEM image of W_18_O_49_ nanowires after anneal.


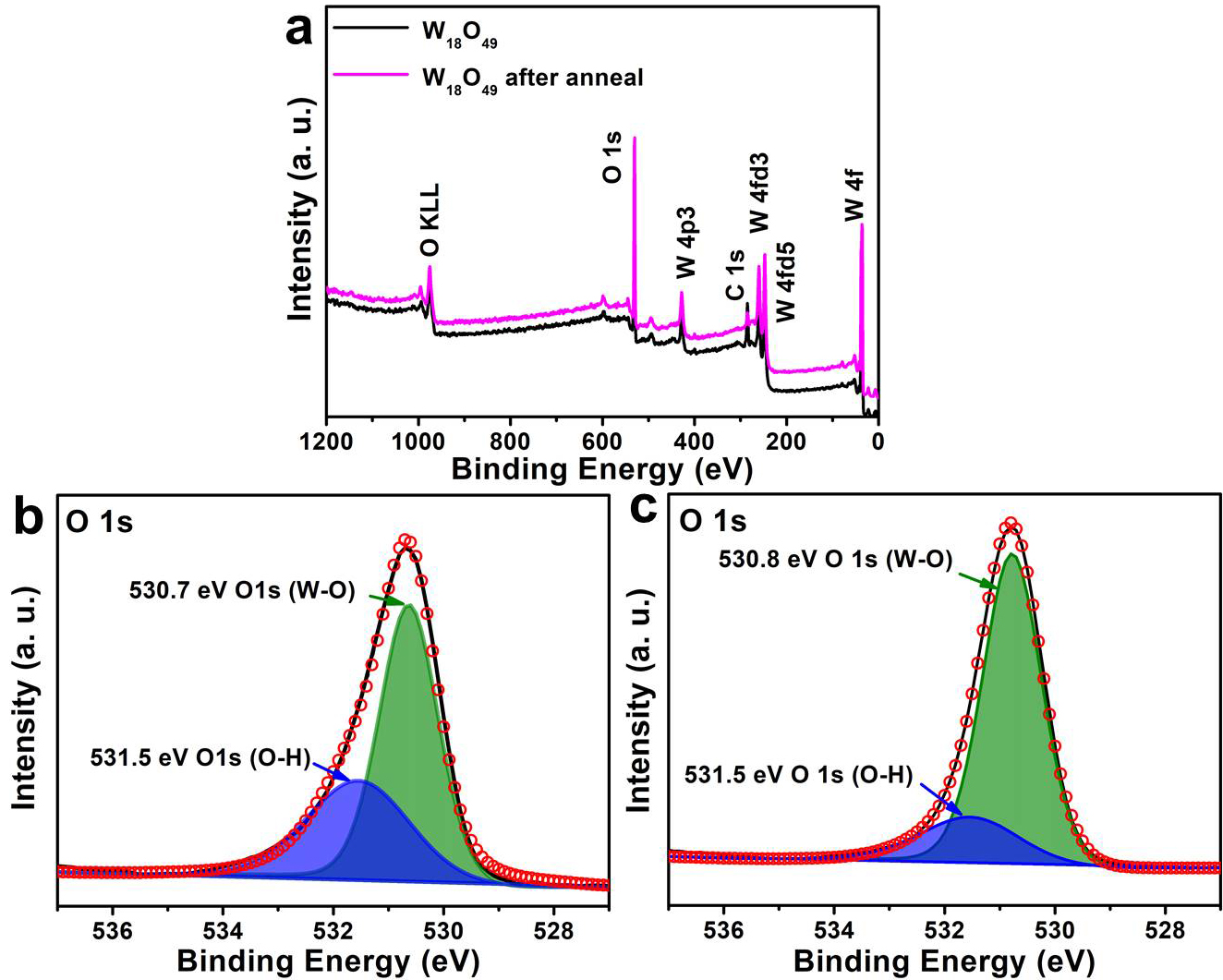


Figure S12. (a) XPS full-range spectra of original and annealed W_18_O_49_ nanowires for 30 min. High-resolution XPS O 1s of (b) W_18_O_49_ nanowires. (c) Annealed W_18_O_49_ nanowires for 30 min.


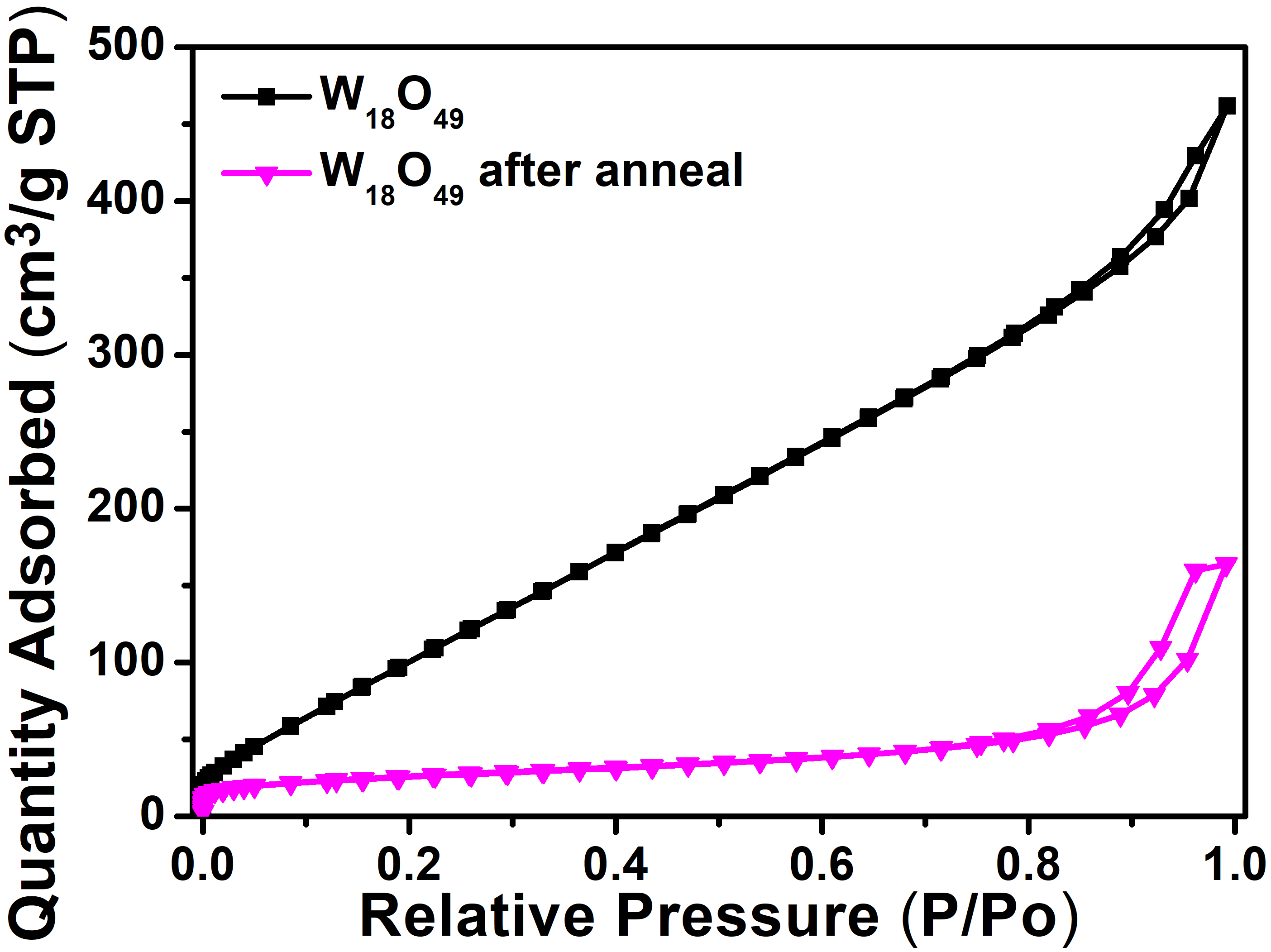


Figure S13. N_2_ adsorption/desorption isotherms at 77 K of W_18_O_49_ nanowires and annealed W_18_O_49_ nanowires.


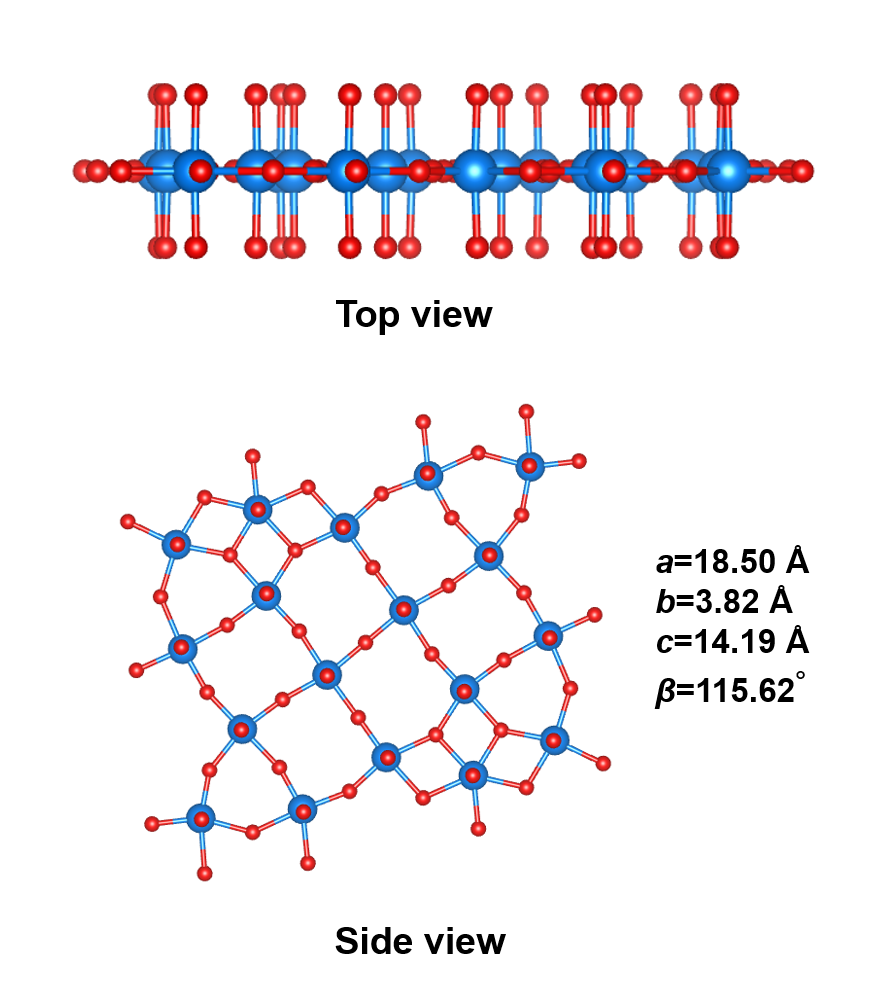


**Figure S14.** The top view, side view and lattice parameters of bulk unit cell of W_18_O_49._


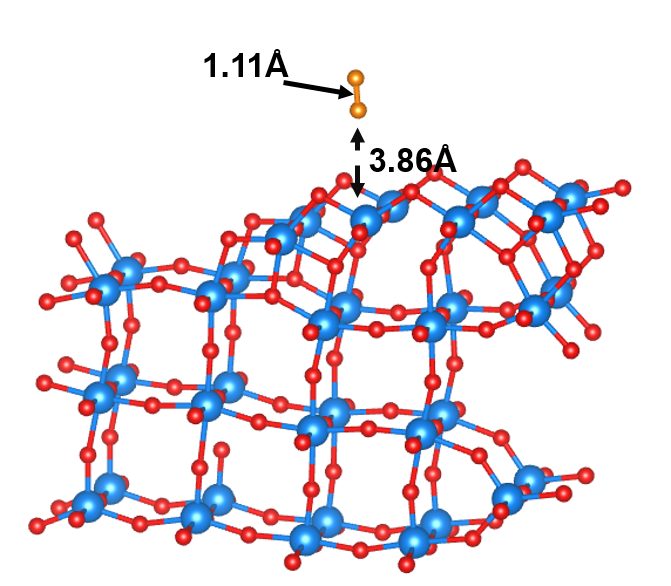


**Figure S15.** The adsorption configuration of N_2_ molecule on the perfect W_18_O_49_ (001) facet without oxygen vacancy.


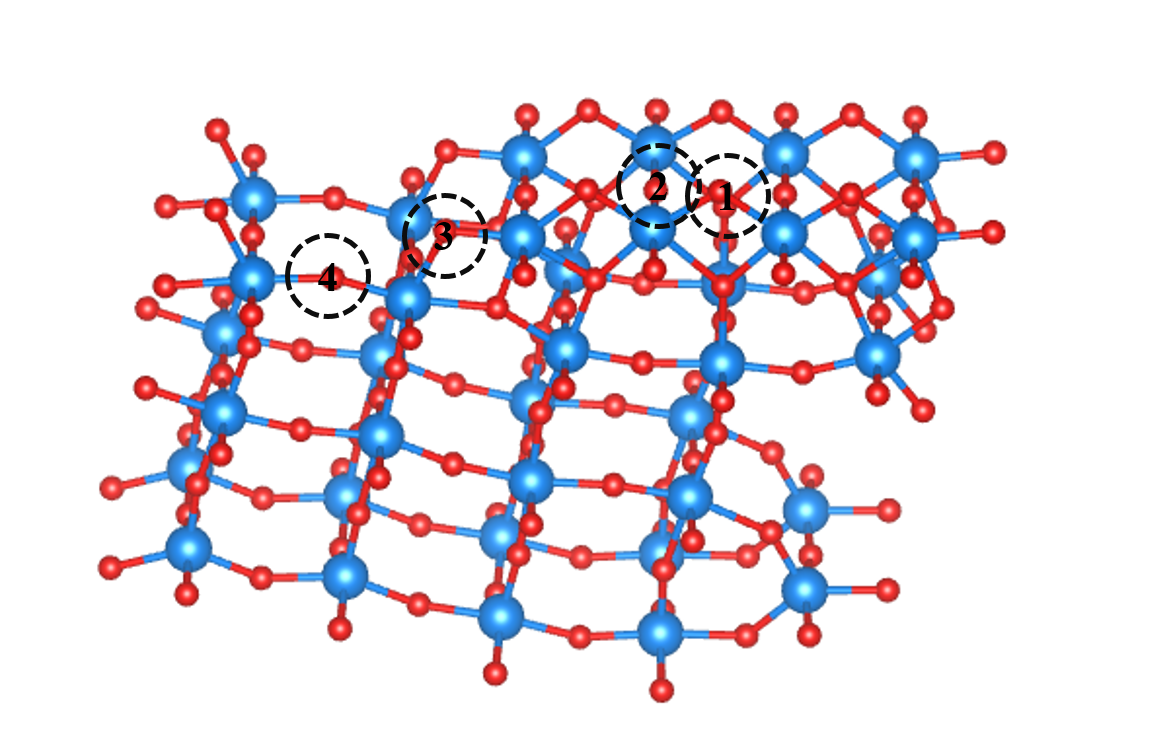


**Figure S16.** The different oxygen vacancies on the W_18_O_49_ nanowires.


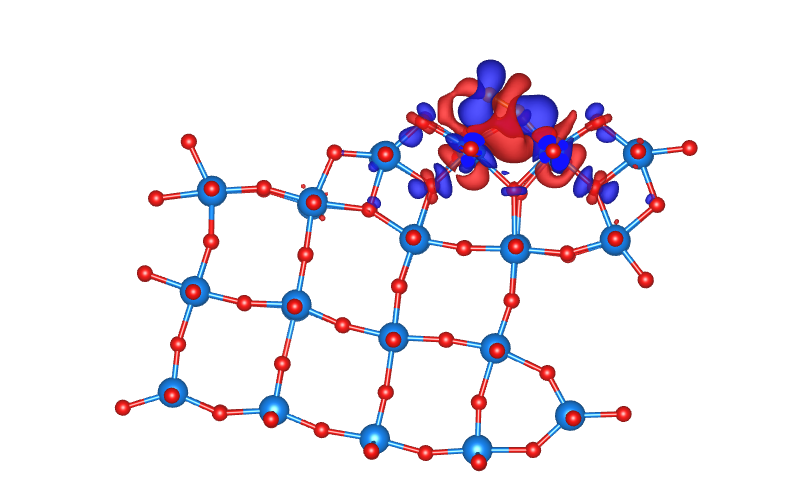


Figure S17. The charge difference analysis of the N_2_ adsorption configuration on the W_18_O_49_ (001) facet with one oxygen vacancy, the blue represent charge accumulation while the red indicate charge depletion.


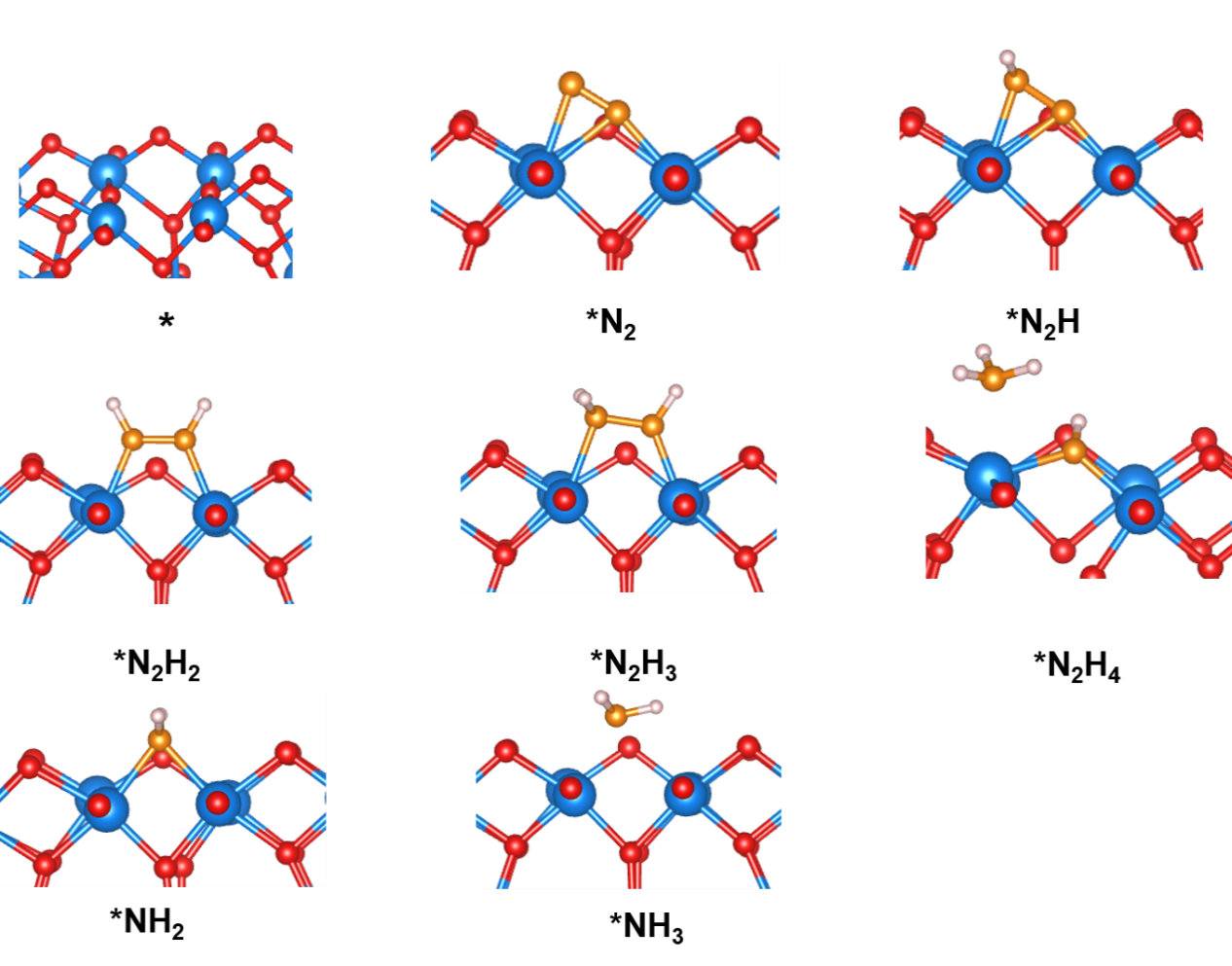


Figure S18. The optimized geometries for reaction intermediates during the nitrogen fixation process to NH_3_ product over W_18_O_49_ (001) with one oxygen vacancy.


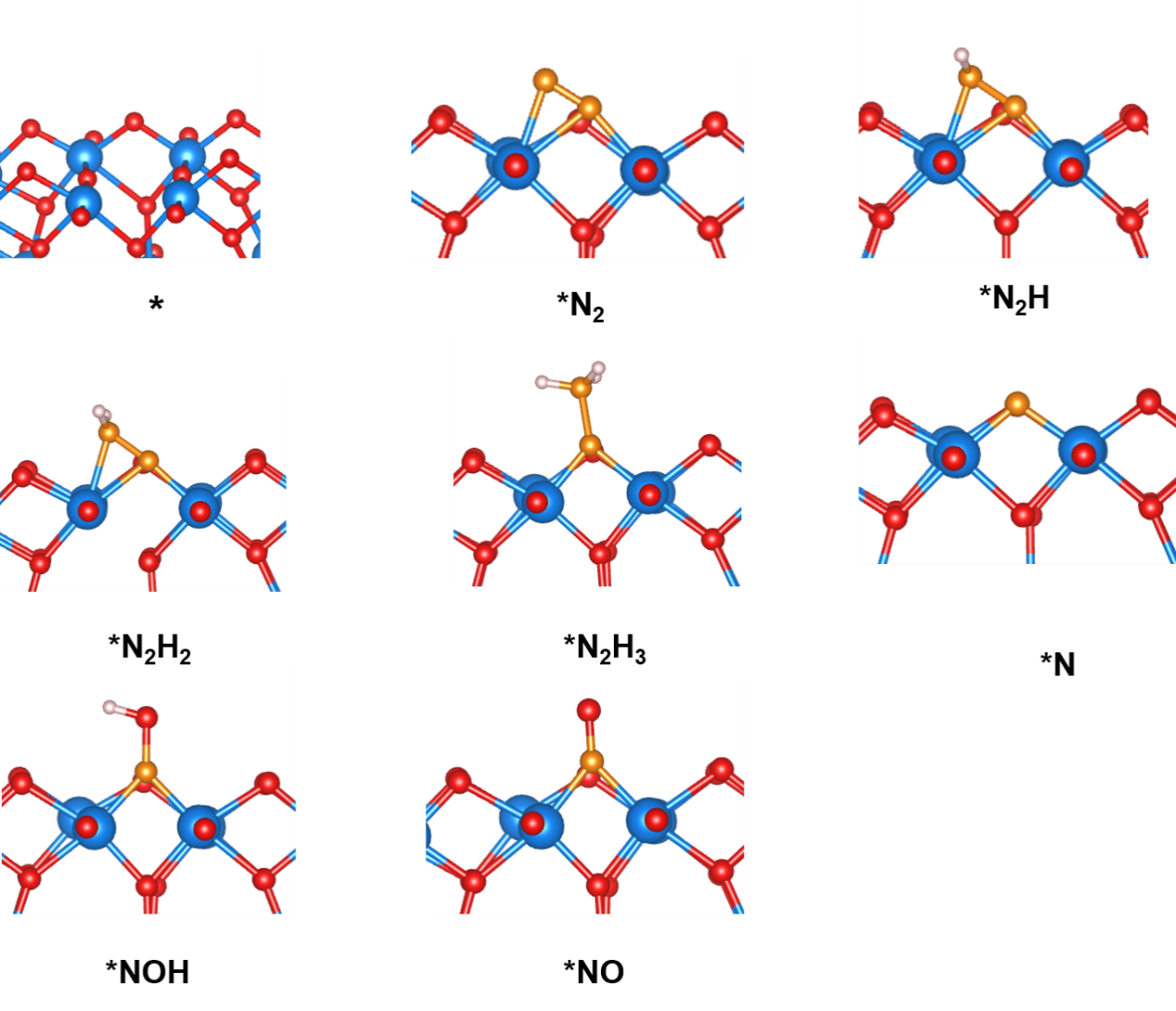


Figure S19. The optimized geometries for reaction intermediates during the nitrogen fixation process to NH_3_ and NO products over W_18_O_49_ (001) with one oxygen vacancy.


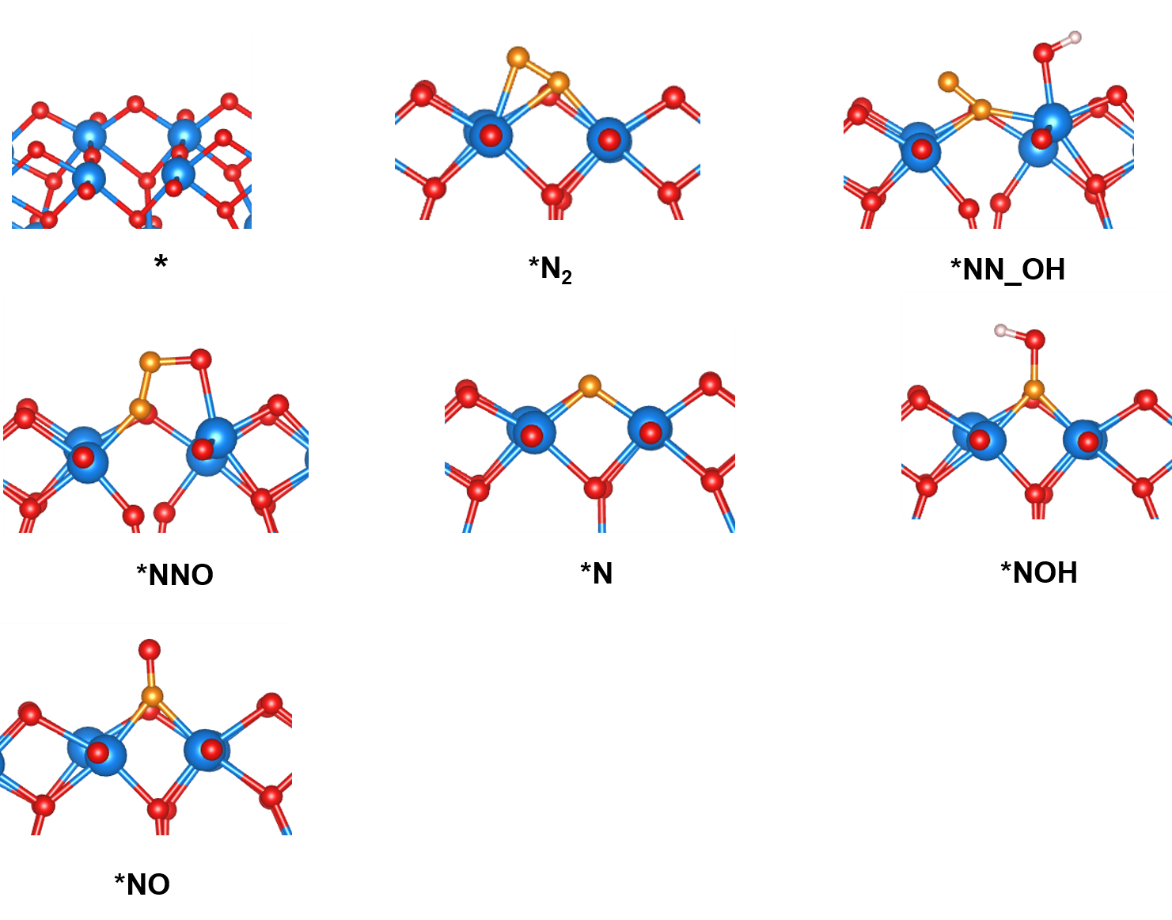


Figure S20. The optimized geometries for reaction intermediates during the nitrogen fixation process to NO product over W_18_O_49_ (001) with one oxygen vacancy.


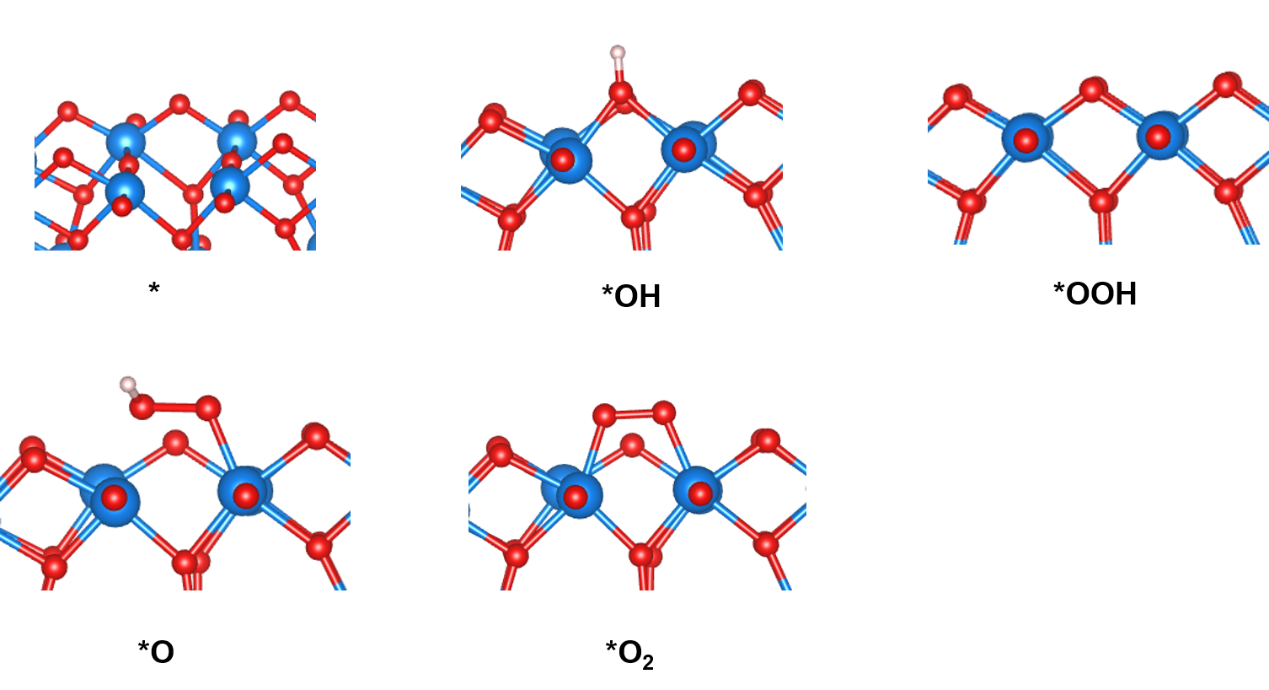


**Figure S21.** The optimized geometries for reaction intermediates during the OER process over W_18_O_49_ (001) with one oxygen vacancy.


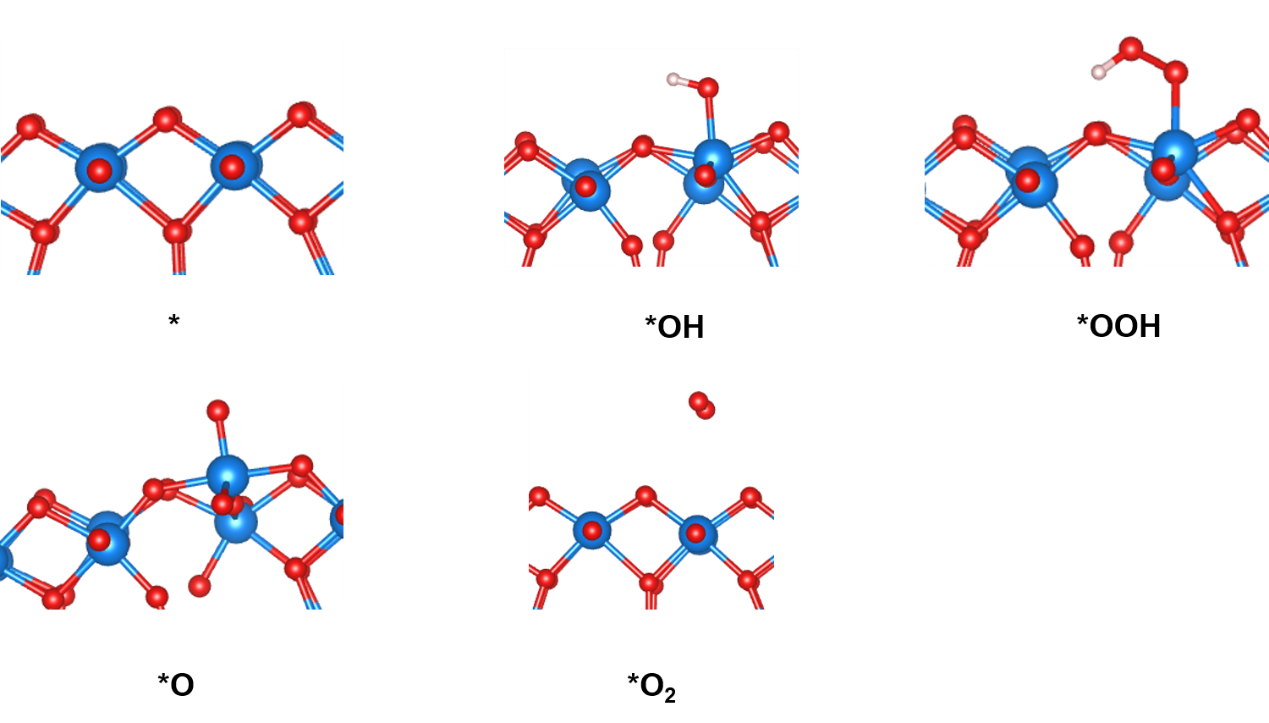


**Figure S22.** The optimized geometries for reaction intermediates during the OER process over the pristin W_18_O_49_ (001).


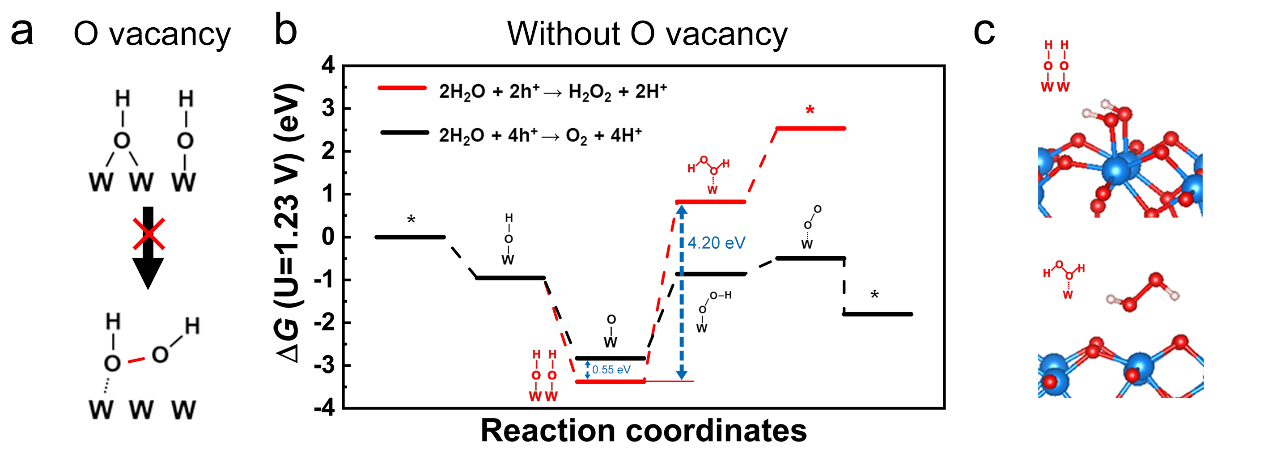


**Figure S23.** Formation of H_2_O_2_ on W_18_O_49_ (001) facet (a) with and (b) without one O vacancy, and (c) the 2*OH and *O_2_H_2_ intermediates on the surface without O vacancy.

When O vacancy is present, the O-O bond in H_2_O_2_ cannot form via the 2*OH intermediate after structural optimization, which is possibly due to the strong O-W bond between the *OH intermediate at O vacancy site and the W atoms around the vacancy. Only after the *OH intermediate at O vacancy site is dehydrogenated can the O-O bond be established, but this corresponds to the formation of *OOH intermediate in the path of OER. When O vacancy is absent, the formation of 2*OH intermediate along the path for producing H_2_O_2_, is energetically more favorable than *O. However, it takes 4.20 eV for the coupling of the two O atoms to form *O_2_H_2_, which is substantially higher than the energy for the hydrogen-transfer reaction 2*OH → *O + H_2_O (0.55 eV). It means that only negligible amount of *O_2_H_2_ intermediates would form on W_18_O_49_ (001) facet. Therefore, the formation of H_2_O_2_ on W_18_O_49_ via oxidation reactions is largely prohibited either with or without an O vacancy. Moreover, if we consider the reduction reaction of O_2_ → H_2_O_2_, we find that the proton is energetically more difficult to attack the W-bonded O atom in *OOH → *O_2_H_2_ reaction (1.69 eV). The proton prefers to attack the remote O atom and release a H_2_O molecule (−1.97 eV), thereby forming the *O intermediate. Overall, our theoretical results strongly suggest that H_2_O_2_ may hardly take part in the reactions of our system.

Table S1. The area of characteristic peak and corresponding ammonia and nitrate concentration

| illuminant | Ammonia | | | | Nitrate | | | |
| --- | --- | --- | --- | --- | --- | --- | --- | --- |
| solar simulator | Time (h) | Retention (min) | Area ((μs/cm)min) | Concentration (ppm) | Time (h) | Retention (min) | Area ((μs/cm)min) | Concentration (ppm) |
|  | 0 | 11.16 | 0.028 | 0.030 | 0 | 12.08 | 0.122 | 0.012 |
|  | 3 | 11.07 | 1.552 | 0.094 | 3 | 12.07 | 0.153 | 0.015 |
|  | 6 | 11.1 | 2.957 | 0.153 | 6 | 12.05 | 0.192 | 0.018 |
|  | 9 | 11.06 | 4.672 | 0.225 | 9 | 12.08 | 0.287 | 0.026 |
|  | 12 | 11.05 | 5.887 | 0.276 | 12 | 12.08 | 0.358 | 0.032 |
| 365 nm LED | Time (h) | Retention (min) | Area ((μs/cm)min) | Concentration (ppm) | Time (h) | Retention (min) | Area ((μs/cm)min) | Concentration (ppm) |
|  | 0 | 11.05 | 0.194 | 0.037 | 0 | 12.130 | 0.110 | 0.011 |
|  | 1 | 11.04 | 0.790 | 0.062 | 1 | 12.210 | 0.119 | 0.012 |
|  | 2 | 11.06 | 1.171 | 0.078 | 2 | 12.120 | 0.146 | 0.014 |
|  | 3 | 11.06 | 2.076 | 0.116 | 3 | 12.150 | 0.252 | 0.023 |
|  | 4 | 11.07 | 2.362 | 0.128 | 4 | 12.170 | 0.370 | 0.033 |
|  | 5 | 11.08 | 2.481 | 0.133 | 5 | 12.160 | 0.453 | 0.040 |
| 384.3 nm LED | Time (h) | Retention (min) | Area ((μs/cm)min) | Concentration (ppm) | Time (h) | Retention (min) | Area ((μs/cm)min) | Concentration (ppm) |
|  | 0 | 11.06 | 0.051 | 0.031 | 0 | 12.1 | 0.098 | 0.010 |
|  | 1 | 11.07 | 0.290 | 0.041 | 1 | 12.11 | 0.146 | 0.014 |
|  | 2 | 11.06 | 0.480 | 0.049 | 2 | 12.08 | 0.264 | 0.024 |
|  | 3 | 11.08 | 0.790 | 0.062 | 3 | 12.12 | 0.299 | 0.027 |
|  | 4 | 11.09 | 0.957 | 0.069 | 4 | 12.11 | 0.370 | 0.033 |
|  | 5 | 11.1 | 1.290 | 0.083 | 5 | 12.09 | 0.393 | 0.035 |
| 400 nm LED | Time (h) | Retention (min) | Area ((μs/cm)min) | Concentration (ppm) | Time (h) | Retention (min) | Area ((μs/cm)min) | Concentration (ppm) |
|  | 0 | 11.23 | 0.051 | 0.031 | 0 | 12.21 | 0.134 | 0.013 |
|  | 1 | 11.26 | 0.456 | 0.048 | 1 | 12.16 | 0.181 | 0.017 |
|  | 2 | 11.32 | 0.599 | 0.054 | 2 | 12.18 | 0.228 | 0.021 |
|  | 3 | 11.17 | 0.695 | 0.058 | 3 | 12.17 | 0.323 | 0.029 |
|  | 4 | 11.14 | 1.028 | 0.072 | 4 | 12.14 | 0.417 | 0.037 |
|  | 5 | 11.15 | 1.171 | 0.078 | 5 | 12.16 | 0.441 | 0.039 |
| 427 nm LED | Time (h) | Retention (min) | Area ((μs/cm)min) | Concentration (ppm) | Time (h) | Retention (min) | Area ((μs/cm)min) | Concentration (ppm) |
|  | 0 | 11.12 | 0.075 | 0.032 | 0 | 12.31 | 0.134 | 0.013 |
|  | 1 | 11.08 | 0.206 | 0.037 | 1 | 12.32 | 0.218 | 0.020 |
|  | 2 | 11.11 | 0.287 | 0.041 | 2 | 12.28 | 0.266 | 0.024 |
|  | 3 | 11.13 | 0.394 | 0.045 | 3 | 12.25 | 0.441 | 0.039 |
|  | 4 | 11.15 | 0.614 | 0.055 | 4 | 12.23 | 0.548 | 0.048 |
|  | 5 | 11.1 | 0.695 | 0.058 | 5 | 12.26 | 0.597 | 0.052 |
| 468.4 nm LED | Time (h) | Retention (min) | Area ((μs/cm)min) | Concentration (ppm) | Time (h) | Retention (min) | Area ((μs/cm)min) | Concentration (ppm) |
|  | 0 | 10.92 | 0.099 | 0.033 | 0 | 12.16 | 0.122 | 0.012 |
|  | 1 | 10.93 | 0.242 | 0.039 | 1 | 12.15 | 0.205 | 0.019 |
|  | 2 | 10.82 | 0.552 | 0.052 | 2 | 12.11 | 0.500 | 0.044 |
|  | 3 | 10.83 | 0.837 | 0.064 | 3 | 12.15 | 0.535 | 0.047 |
|  | 4 | 10.9 | 0.933 | 0.068 | 4 | 12.18 | 0.937 | 0.081 |
|  | 5 | 10.82 | 1.076 | 0.074 | 5 | 12.21 | 1.055 | 0.091 |
| 498 nm LED | Time (h) | Retention (min) | Area ((μs/cm)min) | Concentration (ppm) | Time (h) | Retention (min) | Area ((μs/cm)min) | Concentration (ppm) |
|  | 0 | 11.01 | 0.075 | 0.032 | 0 | 12.19 | 0.098 | 0.010 |
|  | 1 | 11.05 | 0.528 | 0.051 | 1 | 12.18 | 0.181 | 0.017 |
|  | 2 | 11.04 | 0.575 | 0.053 | 2 | 12.21 | 0.216 | 0.020 |
|  | 3 | 11.06 | 0.957 | 0.069 | 3 | 12.22 | 0.382 | 0.034 |
|  | 4 | 11.08 | 1.195 | 0.079 | 4 | 12.25 | 0.559 | 0.049 |
|  | 5 | 11.08 | 1.838 | 0.106 | 5 | 12.23 | 0.677 | 0.059 |
| 515 nm LED | Time (h) | Retention (min) | Area ((μs/cm)min) | Concentration (ppm) | Time (h) | Retention (min) | Area ((μs/cm)min) | Concentration (ppm) |
|  | 0 | 11.16 | 0.078 | 0.032 | 0 | 12.15 | 0.110 | 0.011 |
|  | 1 | 11.07 | 0.259 | 0.040 | 1 | 12.21 | 0.230 | 0.021 |
|  | 2 | 11.1 | 0.411 | 0.046 | 2 | 12.19 | 0.438 | 0.039 |
|  | 3 | 11.23 | 0.673 | 0.057 | 3 | 12.18 | 0.614 | 0.054 |
|  | 4 | 11.18 | 0.914 | 0.067 | 4 | 12.22 | 0.744 | 0.065 |
|  | 5 | 11.16 | 1.007 | 0.071 | 5 | 12.13 | 0.854 | 0.074 |
| 590 nm LED | Time (h) | Retention (min) | Area ((μs/cm)min) | Concentration (ppm) | Time (h) | Retention (min) | Area ((μs/cm)min) | Concentration (ppm) |
|  | 0 | 11.04 | 0.051 | 0.031 | 0 | 12.21 | 0.098 | 0.010 |
|  | 1 | 11.09 | 0.194 | 0.037 | 1 | 12.23 | 0.122 | 0.012 |
|  | 2 | 11.15 | 0.361 | 0.044 | 2 | 12.2 | 0.181 | 0.017 |
|  | 3 | 11.12 | 0.599 | 0.054 | 3 | 12.19 | 0.193 | 0.018 |
|  | 4 | 11.11 | 0.671 | 0.057 | 4 | 12.18 | 0.205 | 0.019 |
|  | 5 | 11.19 | 1.291 | 0.080 | 5 | 12.21 | 0.240 | 0.022 |
| 620 nm LED | Time (h) | Retention (min) | Area ((μs/cm)min) | Concentration (ppm) | Time (h) | Retention (min) | Area ((μs/cm)min) | Concentration (ppm) |
|  | 0 | 11.02 | 0.051 | 0.032 | 0 | 12.15 | 0.110 | 0.011 |
|  | 1 | 11.03 | 0.194 | 0.036 | 1 | 12.21 | 0.114 | 0.011 |
|  | 2 | 11.02 | 0.361 | 0.042 | 2 | 12.23 | 0.122 | 0.012 |
|  | 3 | 11.07 | 0.599 | 0.046 | 3 | 12.18 | 0.134 | 0.013 |
|  | 4 | 11.06 | 0.671 | 0.050 | 4 | 12.19 | 0.146 | 0.013 |
|  | 5 | 11.12 | 1.219 | 0.057 | 5 | 12.23 | 0.151 | 0.014 |
| 730 nm LED | Time (h) | Retention (min) | Area ((μs/cm)min) | Concentration (ppm) | Time (h) | Retention (min) | Area ((μs/cm)min) | Concentration (ppm) |
|  | 0 | 11.24 | 0.099 | 0.033 | 0 | 12.08 | 0.098 | 0.010 |
|  | 1 | 11.19 | 0.228 | 0.038 | 1 | 12.09 | 0.126 | 0.012 |
|  | 2 | 11.25 | 0.354 | 0.044 | 2 | 12.11 | 0.151 | 0.014 |
|  | 3 | 11.22 | 0.418 | 0.046 | 3 | 12.13 | 0.185 | 0.017 |
|  | 4 | 11.21 | 0.504 | 0.050 | 4 | 12.15 | 0.222 | 0.020 |
|  | 5 | 11.19 | 0.580 | 0.053 | 5 | 12.09 | 0.254 | 0.023 |
| 850 nm LED | Time (h) | Retention (min) | Area ((μs/cm)min) | Concentration (ppm) | Time (h) | Retention (min) | Area ((μs/cm)min) | Concentration (ppm) |
|  | 0 | 0 | 0 | 0 | 0 | 0 | 0 | 0.000 |
|  | 1 | 0 | 0 | 0 | 1 | 0 | 0 | 0.000 |
|  | 2 | 0 | 0 | 0 | 2 | 0 | 0 | 0.000 |
|  | 3 | 0 | 0 | 0 | 3 | 0 | 0 | 0.000 |
|  | 4 | 0 | 0 | 0 | 4 | 0 | 0 | 0.000 |
|  | 5 | 0 | 0 | 0 | 5 | 0 | 0 | 0.000 |
| Xenon lamp | Time (h) | Retention (min) | Area ((μs/cm)min) | Concentration (ppm) | Time (h) | Retention (min) | Area ((μs/cm)min) | Concentration (ppm) |
|  | 0 | 11.19 | 0.051 | 0.031 | 0 | 13.18 | 0.098 | 0.010 |
|  | 1 | 11.24 | 1.245 | 0.081 | 1 | 13.14 | 0.117 | 0.012 |
|  | 2 | 11.25 | 3.036 | 0.156 | 2 | 12.78 | 0.145 | 0.014 |
|  | 3 | 11.22 | 4.129 | 0.202 | 3 | 12.72 | 0.161 | 0.015 |
|  | 4 | 11.21 | 5.744 | 0.270 | 4 | 12.76 | 0.176 | 0.017 |
|  | 5 | 11.19 | 6.363 | 0.296 | 5 | 13.01 | 0.192 | 0.018 |
| Xenon lamp（annealed W_18_O_49_） | Time (h) | Retention (min) | Area ((μs/cm)min) | Concentration (ppm) | Time (h) | Retention (min) | Area ((μs/cm)min) | Concentration (ppm) |
|  | 0 | 11.1 | 0.075 | 0.032 | 0 | 13.01 | 0.122 | 0.012 |
|  | 1 | 11.12 | 0.099 | 0.033 | 1 | 13.14 | 0.122 | 0.012 |
|  | 2 | 11.15 | 0.171 | 0.036 | 2 | 13.15 | 0.122 | 0.012 |
|  | 3 | 11.16 | 0.218 | 0.038 | 3 | 13.18 | 0.122 | 0.012 |
| Xenon lamp Run1 | Time (h) | Retention (min) | Area ((μs/cm)min) | Concentration (ppm) | Time (h) | Retention (min) | Area ((μs/cm)min) | Concentration (ppm) |
|  | 0 | 10.92 | 0.075 | 0.032 | 0 | 13.19 | 0.110 | 0.011 |
|  | 3 | 11.01 | 0.600 | 0.096 | 3 | 13.19 | 0.134 | 0.013 |
|  | 6 | 10.95 | 3.005 | 0.155 | 6 | 13.29 | 0.169 | 0.016 |
|  | 9 | 10.96 | 4.720 | 0.227 | 9 | 13.21 | 0.275 | 0.025 |
|  | 12 | 11.05 | 5.935 | 0.278 | 12 | 13.22 | 0.346 | 0.031 |
| Xenon lamp Run 2 | Time (h) | Retention (min) | Area ((μs/cm)min) | Concentration (ppm) | Time (h) | Retention (min) | Area ((μs/cm)min) | Concentration (ppm) |
|  | 0 | 11.01 | 0.099 | 0.033 | 0 | 13.18 | 0.098 | 0.010 |
|  | 3 | 11.02 | 1.600 | 0.096 | 3 | 13.17 | 0.234 | 0.022 |
|  | 6 | 11.05 | 3.100 | 0.159 | 6 | 13.19 | 0.258 | 0.024 |
|  | 9 | 11.1 | 4.696 | 0.226 | 9 | 13.22 | 0.287 | 0.026 |
|  | 12 | 11.09 | 5.985 | 0.279 | 12 | 13.21 | 0.317 | 0.028 |
| Xenon lamp Run 3 | Time (h) | Retention (min) | Area ((μs/cm)min) | Concentration (ppm) | Time (h) | Retention (min) | Area ((μs/cm)min) | Concentration (ppm) |
|  | 0 | 11.16 | 0.051 | 0.031 | 0 | 13.12 | 0.098 | 0.010 |
|  | 3 | 11.07 | 1.590 | 0.096 | 3 | 13.09 | 0.140 | 0.013 |
|  | 6 | 11.08 | 2.910 | 0.151 | 6 | 13.11 | 0.181 | 0.017 |
|  | 9 | 11.18 | 4.291 | 0.209 | 9 | 13.15 | 0.258 | 0.024 |
|  | 12 | 11.12 | 5.673 | 0.267 | 12 | 13.16 | 0.311 | 0.028 |
| Xenon lamp Run 4 | Time (h) | Retention (min) | Area ((μs/cm)min) | Concentration (ppm) | Time (h) | Retention (min) | Area ((μs/cm)min) | Concentration (ppm) |
|  | 0 | 11.05 | 0.075 | 0.032 | 0 | 13.11 | 0.098 | 0.010 |
|  | 3 | 11.04 | 1.457 | 0.090 | 3 | 13.23 | 0.228 | 0.021 |
|  | 6 | 11.06 | 2.743 | 0.144 | 6 | 13.21 | 0.334 | 0.030 |
|  | 9 | 11.08 | 4.005 | 0.197 | 9 | 13.16 | 0.393 | 0.035 |
|  | 12 | 11.05 | 5.606 | 0.264 | 12 | 13.19 | 0.441 | 0.039 |
| Xenon lamp Run 5 | Time (h) | Retention (min) | Area ((μs/cm)min) | Concentration (ppm) | Time (h) | Retention (min) | Area ((μs/cm)min) | Concentration (ppm) |
|  | 0 | 11.13 | 0.075 | 0.032 | 0 | 13.17 | 0.110 | 0.011 |
|  | 3 | 11.16 | 1.504 | 0.092 | 3 | 13.19 | 0.240 | 0.022 |
|  | 6 | 11.12 | 2.862 | 0.149 | 6 | 13.15 | 0.358 | 0.032 |
|  | 9 | 11.11 | 4.101 | 0.201 | 9 | 13.13 | 0.405 | 0.036 |
|  | 12 | 11.14 | 5.649 | 0.266 | 12 | 13.16 | 0.429 | 0.038 |
| Xenon lamp Run 6 | Time (h) | Retention (min) | Area ((μs/cm)min) | Concentration (ppm) | Time (h) | Retention (min) | Area ((μs/cm)min) | Concentration (ppm) |
|  | 0 | 11.05 | 0.075 | 0.031 | 0 | 13.21 | 0.122 | 0.012 |
|  | 3 | 11.06 | 1.504 | 0.088 | 3 | 13.19 | 0.264 | 0.024 |
|  | 6 | 11.07 | 2.682 | 0.132 | 6 | 13.23 | 0.323 | 0.029 |
|  | 9 | 11.12 | 4.101 | 0.195 | 9 | 13.18 | 0.370 | 0.033 |
|  | 12 | 11.16 | 5.649 | 0.258 | 12 | 13.21 | 0.417 | 0.037 |
| Xenon lamp Run 7 | Time (h) | Retention (min) | Area ((μs/cm)min) | Concentration (ppm) | Time (h) | Retention (min) | Area ((μs/cm)min) | Concentration (ppm) |
|  | 0 | 11.11 | 0.075 | 0.032 | 0 | 13.15 | 0.122 | 0.012 |
|  | 3 | 11.12 | 1.504 | 0.092 | 3 | 13.16 | 0.252 | 0.023 |
|  | 6 | 11.08 | 2.862 | 0.149 | 6 | 13.17 | 0.370 | 0.033 |
|  | 9 | 11.09 | 4.101 | 0.201 | 9 | 13.15 | 0.417 | 0.037 |
|  | 12 | 11.04 | 5.701 | 0.268 | 12 | 13.21 | 0.441 | 0.039 |
| Xenon lamp Run 8 | Time (h) | Retention (min) | Area ((μs/cm)min) | Concentration (ppm) | Time (h) | Retention (min) | Area ((μs/cm)min) | Concentration (ppm) |
|  | 0 | 11.16 | 0.099 | 0.033 | 0 | 13.19 | 0.110 | 0.011 |
|  | 3 | 11.12 | 1.481 | 0.091 | 3 | 13.25 | 0.240 | 0.022 |
|  | 6 | 11.15 | 2.767 | 0.145 | 6 | 13.21 | 0.346 | 0.031 |
|  | 9 | 11.14 | 4.072 | 0.200 | 9 | 13.17 | 0.405 | 0.036 |
|  | 12 | 11.12 | 5.606 | 0.264 | 12 | 13.18 | 0.453 | 0.040 |
| Xenon lamp Run 9 | Time (h) | Retention (min) | Area ((μs/cm)min) | Concentration (ppm) | Time (h) | Retention (min) | Area ((μs/cm)min) | Concentration (ppm) |
|  | 0 | 11.26 | 0.075 | 0.032 | 0 | 13.26 | 0.098 | 0.010 |
|  | 3 | 11.23 | 1.657 | 0.098 | 3 | 13.29 | 0.140 | 0.013 |
|  | 6 | 11.21 | 2.891 | 0.150 | 6 | 13.25 | 0.181 | 0.017 |
|  | 9 | 11.32 | 4.272 | 0.208 | 9 | 13.27 | 0.258 | 0.024 |
|  | 12 | 11.17 | 5.804 | 0.272 | 12 | 13.21 | 0.311 | 0.028 |
| Xenon lamp Run 10 | Time (h) | Retention (min) | Area ((μs/cm)min) | Concentration (ppm) | Time (h) | Retention (min) | Area ((μs/cm)min) | Concentration (ppm) |
|  | 0 | 11.14 | 0.075 | 0.032 | 0 | 13.19 | 0.098 | 0.010 |
|  | 3 | 11.17 | 1.409 | 0.088 | 3 | 13.21 | 0.244 | 0.022 |
|  | 6 | 11.21 | 2.502 | 0.134 | 6 | 13.22 | 0.299 | 0.027 |
|  | 9 | 11.23 | 4.046 | 0.199 | 9 | 13.23 | 0.346 | 0.031 |
|  | 12 | 11.26 | 5.568 | 0.263 | 12 | 13.18 | 0.393 | 0.035 |

**Table S2**. The delta value of atomic percentage of ^18^O.

| **Peak** | **d ^18^O/^16^O [per mil] vs.**  **VSMOW** | **^18^O/O (%)** |
| --- | --- | --- |
| **Peak** 1 | 34.350 | 0.206979 |
| **Peak** 2 | 34.118 | 0.206932 |
| **Peak** 3 | 34.060 | 0.206921 |
| **Peak** 4 | 10.524 | 0.202221 |

Table S3. The formation energy of different oxygen vacancy

| O vacancy | Formation energy (eV) |
| --- | --- |
| 1 | 4.47 |
| 2 | 5.29 |
| 3 | 4.51 |
| 4 | 5.59 |

**References**

1. G. Kresse, D. Joubert, From ultrasoft pseudopotentials to the projector augmented-wave method. *Phys. Rev. B*. **59**, 1758–1775 (1999).

2. F. Han, Projector augmented-wave method. *Phys. Rev. B*. **50**, 17953–17979 (1994).

3. J. P. Perdew, J. A. Chevary, S. H. Vosko, K. A. Jackson, M. R. Pederson, D. J. Singh Atoms, molecules, solids, and surfaces: Applications of the generalized gradient approximation for exchange and correlation. *Phys. Rev. B*. **46**, 6671–6687 (1992).

4. J. Rossmeisl, Z. W. Qu, H. Zhu, G. J. Kroes, J. K. Nørskov, Electrolysis of water on oxide surfaces. *J. Electroanal. Chem.* **607**, 83–89 (2007).
